# The Fgf/Erf/NCoR1/2 repressive axis controls trophoblast cell fate

**DOI:** 10.1038/s41467-023-38101-8

**Published:** 2023-05-04

**Authors:** Andreas Lackner, Michael Müller, Magdalena Gamperl, Delyana Stoeva, Olivia Langmann, Henrieta Papuchova, Elisabeth Roitinger, Gerhard Dürnberger, Richard Imre, Karl Mechtler, Paulina A. Latos

**Affiliations:** 1 Center for Anatomy and Cell Biology, Medical University of Vienna, A-1090 Vienna, Austria; 2 Institute of Molecular Pathology, A-1030 Vienna, Austria

**Keywords:** Stem-cell differentiation, Epigenetics, Epigenetics

## Abstract

Placental development relies on coordinated cell fate decisions governed by signalling inputs. However, little is known about how signalling cues are transformed into repressive mechanisms triggering lineage-specific transcriptional signatures. Here, we demonstrate that upon inhibition of the Fgf/Erk pathway in mouse trophoblast stem cells (TSCs), the Ets2 repressor factor (Erf) interacts with the Nuclear Receptor Co-Repressor Complex 1 and 2 (NCoR1/2) and recruits it to key trophoblast genes. Genetic ablation of *Erf* or *Tbl1x* (a component of the NCoR1/2 complex) abrogates the Erf/NCoR1/2 interaction. This leads to mis-expression of Erf/NCoR1/2 target genes, resulting in a TSC differentiation defect. Mechanistically, Erf regulates expression of these genes by recruiting the NCoR1/2 complex and decommissioning their H3K27ac-dependent enhancers. Our findings uncover how the Fgf/Erf/NCoR1/2 repressive axis governs cell fate and placental development, providing a paradigm for Fgf-mediated transcriptional control.

## Abstract (web summary)

Repression of gene expression contributes to lineage-specific transcriptional signatures. Here Lackner et al. demonstrate that the Erf-NCoR1/2 complex controls trophoblast differentiation by linking signalling with transcriptional repression

## Introduction

**Fig. 1:**
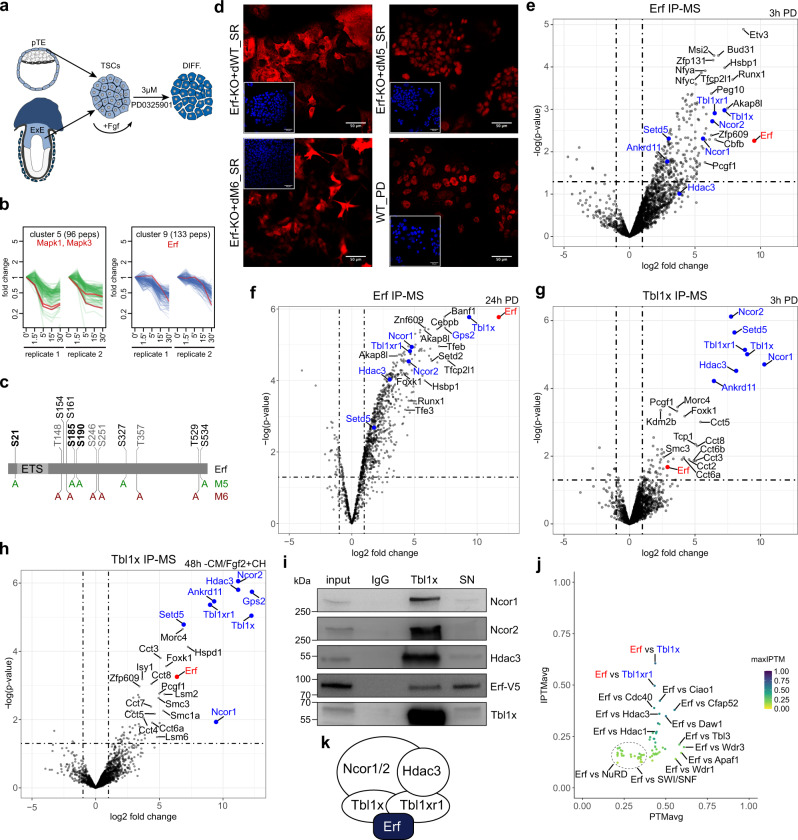
Erf interacts with NCoR1/2 complexes in differentiating TSCs. **a** A diagram showing that trophoblast stem cells (TSCs) can be derived from the polar trophectoderm (pTE) and the extraembryonic ectoderm (ExE) of the mouse embryo and self-renew in the presence of Fgf. Treatment with the Mek inhibitor PD0325901 (PD) results in exiting self-renewal and their differentiation (DIFF.). **b** Phosphoproteomic analysis of TSCs treated for 1.5, 5, 15, and 30 min with 3 µM Mek inhibitor, compared to the untreated (0') and displayed as fold change. Analysis based on two biological replicates (*n* = 2). Representative clusters 5 and 9 are shown. Mapk1 and Mapk3 serve as positive controls. **c** Phosphorylation sites found in the Erf protein. Sites identified in our phosphoproteome dataset are depicted in black, those regulated are additionally bolded. Sites that were mutated in the phosphomutants are indicated in green (M5 construct) and red (M6 construct). **d** Confocal images (representative of two biological replicates *n* = 2) of Erf-KO cell lines carrying doxycycline (dox) inducible transgenes of Erf WT, M5 (S21A, S185A, S190A, S534A, S327A) and M6 (S161A, T529A, S246A, S251A, T357A, T148A) phospho-mutants cultured in SR conditions in the presence of (+d) dox. WT cells differentiated in PD serve as positive control. Erf in red, DAPI in inset in blue. **e**, **f** Erf interactomes identified by mass spectrometry after 3 h (**e**) and 24 h (**f**) PD treatment in differentiating TSCs expressing Erf-3xFlag and compared to an empty vector control line (*n* = 3, each). Erf is marked in red, components of the NCoR1/2 complex in blue. Each plot represents three biological replicates of the Erf-3xFlag (*n* = 3) and the empty vector (*n* = 3) pair. **g**, **h** Tbl1x interactomes identified by mass spectrometry after 3 h PD (**g**) and 48 h CM/Fgf withdrawal and CH treatment (**h**) in differentiating TSCs expressing Tbl1x-3xFlag and compared to an empty vector control line (*n* = 3, each). Erf is marked in red, components of the NCoR1/2 complex in blue. Each plot represents three biological replicates of the Tbl1x-3xFlag (*n* = 3) and the empty vector (*n* = 3) pair. **i** Endogenous Tbl1x immunoprecipitates analysed by Western blot with indicated antibodies. IgG serves as a negative control; SN: supernatant. Representative of three biological replicates (*n* = 3). **j** AlphaFold2-Multimer protein interaction plot for Erf as bait and NCoR1/2, SWI/SNF, NuRD complex components, and WD40 proteins showing the iPTM (interface) score for a likely interaction and the PTM (predicted TM) score reflecting the global predicted structure accuracy. **k** A diagram indicating that Erf interacts with the NCoR1/2 complex.

The placenta is the site of exchange of nutrients, oxygen, hormones, metabolic by-products, and other molecules between the maternal and foetal bloodstreams. This exchange is enabled and facilitated by a variety of highly specialised types of trophoblasts that in the mouse include syncytiotrophoblast (SynT-I and SynT-II), spongiotrophoblast (SpT) and a range of trophoblast giant cells (TGCs) among others^1^. They all arise from the trophectoderm, and their developmental specification relies on temporarily and spatially coordinated transcriptional outputs driven by signalling inputs. In particular, the Fgf/Raf/Mek/Erk signalling pathway is indispensable for early placental development, as demonstrated by numerous gene knockouts and its necessity to sustain trophoblast stem cells (TSCs)^2–5^. TSCs represent the extraembryonic ectoderm compartment of the mouse embryo and self-renew in the presence of Fgf4 and Nodal/Activin signalling, while sustaining an undifferentiated, multipotent state. The withdrawal of these components leads to TSCs exiting multipotency and to their differentiation into the various trophoblast cell types of the placenta^5^ (Fig. 1a). However, the underlying mechanisms of transcriptional regulation that silence the multipotent state and activate specific differentiation programmes remain largely unknown.

The Fgf/Raf/Mek/Erk pathway regulates cell morphology, migration, metabolism, proliferation, differentiation, as well as survival and is transduced by a reversible phosphorylation cascade^6^. In the trophoblast context, this pathway is thought to drive a network of transcription factors (TFs) to ensure coordinated placental development. We previously identified *Esrrb* as an early Fgf/Raf/Mek/Erk transcriptional target that directly regulates a group of crucial downstream TFs, including *Eomes* and *Elf5*^7^. Another TF with placental function downstream of the Fgf/Raf/Mek/Erk, and its direct phosphorylation target, is the Ets2 repressor factor (Erf). Erf phosphorylation determines its cellular localization: phosphorylated Erf is predominantly cytoplasmic, while unphosphorylated Erf is nuclear and acts as a transcriptional repressor^8,9^. Importantly, homozygous deletion of *Erf* in mice leads to a block of chorionic differentiation, resulting in the absence of the placental labyrinth layer (including SynT-I and SynT-II) and consequently in embryonic death by E10.5^10^. This phenotype is recapitulated in vitro, as TSCs deficient for *Erf* exhibit delayed differentiation and prolonged expression of self-renewal markers (*Esrrb*, *Cdx2*, *Eomes*); however, the exact mechanism of Erf-dependent gene regulation remains to be elucidated^10^.

Despite the identification of the critical signalling pathways and the TFs that drive TSC self-renewal, the mechanisms that link those factors to specific transcriptional outputs remain poorly understood. In particular, it is unclear how Fgf/Erk signalling brings about the activation of the key TSC TF genes and conversely, how TSCs exit multipotency upon attenuation of this pathway. In general, the exit from multipotency/pluripotency of stem cells and progenitors encompasses the rapid shutdown of their transcriptional programmes—a prerequisite for activation of specification and differentiation programmes. This prompt silencing of genes is often brought about by repressors that recruit transcriptional co-repressor protein complexes. How signalling controls this process is understudied and in the trophoblast context remains unclear. Here, we use the TSC model to dissect the molecular events accompanying the exit from multipotency and discover the Fgf/Erf/NCoR1/2 axis bridging signalling and transcription in early trophoblast cell fate decisions.

## Results

### Erf interacts with NCoR1/2 complexes in TSCs

While Fgf/Erk signalling is indispensable for TSC multipotency, the downstream molecular events are largely unknown. To identify the protein phosphorylation targets of Fgf/Erk signalling, we determined the phospho-proteomic profiles of TSCs during a time-course (1.5, 5, 15, and 30 min) of Mek inhibition using PD0325901 (PD). The analysis revealed 28 peptide clusters that exhibited distinct phosphorylation dynamics upon Mek inhibition (Fig. 1b, Supplementary Fig. 1a and Supplementary Data 1). Cluster 5 contains Mapk1 (Erk2) and Mapk3 (Erk1), the direct Mek targets, that serve as positive controls and thereby validate our results (Fig. 1b). Among other proteins (e.g. Etv5, Bbx, Ybc), in cluster 9 we identified five regulated peptides of Erf phosphorylated on three different serine residues (S21, S185, S190), confirming Erf as one of the early responding phosphorylation targets of Fgf/Erk^8^ (Fig. 1b). In addition, we uncovered phosphorylation at the S154, S161, S185, S190, S327, S534, and T529 residues, without detectable regulation (Supplementary Data 1). In agreement with previous reports^8^, in the presence of Fgf, Erf is predominantly cytoplasmic, yet upon attenuation of Fgf signalling the dephosphorylated Erf becomes nuclear (Supplementary Fig. 1b; for an overview of Erf phosphosites, see www.phosphosite.org). To assess the functional relevance of the Erf phosphorylation sites, we generated two constructs (M5 and M6), where we mutated serine/threonine residues into alanine (Fig. 1c). The set of mutations in M5 (S21A, S185A, S190A, S534A, S327A; conserved in human) has not been tested before while the human mutations in M6 (S161A, T529A, S246A, S251A, T357A, T148A) have been tested previously in fibroblasts^8^. Inducible expression of M5, M6, and WT Erf in Erf-KO TSCs cultured in self-renewal (SR) conditions (in the presence of Fgf) revealed cytoplasmic localization of WT and occasional nuclear localization of the M6 Erf (Fig. 1d). Strikingly, the M5 Erf construct exhibited aberrant nuclear localization despite the presence of Fgf, resulting in reduced expression of key Fgf target genes, including *Esrrb* and *Cdx2* (Supplementary Fig. 1c). Thus, our findings highlight the functional importance of these Erf phospho-residues. Taken together, we identified novel and functionally relevant phosphorylation sites that determine Fgf-dependent cellular localization of Erf in TSCs.

Although Erf was proposed to act as a phosphorylation-dependent repressor in TSCs, the molecular mechanism as to how it brings about transcriptional silencing remains elusive. To address the Erf mode of action, we first set out to determine Erf-interacting partners in TSCs. We stably expressed 3xFlag-tagged Erf (Supplementary Fig. 1d), treated the TSCs with PD for 3 h and 24 h and performed anti-Flag co-immunoprecipitation followed by mass spectrometry. Strikingly, the Erf interactome featured core components of the NCoR1/2 co-repressor complex: Nuclear receptor co-repressor 1 (Ncor1), Nuclear receptor co-repressor 2 (Ncor2), histone deacetylase 3 (Hdac3), transducin (beta)-like 1 X-linked (Tbl1x), transducin (beta)-like 1 X-linked receptor 1 (Tbl1xr1), and G protein pathway suppressor 2 (Gps2) but no other protein complexes (Fig. 1e, f). We also detected the Setd5 and Ankrd11 proteins that frequently co-purify with the NCoR1/2 complex in other systems^11,12^. NCoR1 and NCoR2 (also referred to as SMRT) are highly related complexes classified by the presence of Ncor1 and Ncor2, respectively, and a number of core shared subunits: Tbl1x, Tbl1xr1, Hdac3, and Gps2^13,14^. The NCoR1/2 complex associates with and is recruited by a plethora of TFs, including numerous nuclear receptors (NRs), functions as their co-repressor and brings about transcriptional gene silencing.

To verify the previously unknown Erf/NCoR1/2 interaction, we generated a stable Tbl1x-3xFlag TSC line (Supplementary Fig. 1e) and induced differentiation either by 3 h of PD administration or by 48 h withdrawal of Fgf and conditioned media (CM) combined with CHIR99021 (CH) treatment, the latter driving SynT-II identity^15^. The anti-Flag co-immunoprecipitation followed by mass spectrometry analysis confirmed Erf, the NCoR1/2 components Ncor1, Ncor2, Tbl1xr1, Hdac3, Gps2, Setd5, and Ankrd11 as Tbl1x interactors (Fig. 1g, h). We also detected multiple subunits (Cct2, Cct3, Cct5, Cct6a, Cct6b, Cct7, Cct8, and Tcp1) of the Chaperonin Containing Tcp-1 complex (CCT), reported to be required for the assembly of the NCoR2 complex^16^. The Erf/NCoR1/2 interaction was further corroborated by Tbl1x co-immunoprecipitation of the endogenous complex (Fig. 1i). To independently validate these findings, we employed the recently developed AlphaFold2 (Multimer) structural modelling tool that revolutionised structural predictions^17–20^. We found that the highest average interaction (iPTM) score with Erf as bait was reached by Tbl1x and its homologue Tbl1xr1, reflecting a potential interaction via its WD40 domain (with low Predicted Aligned Error, PAE), whereas other NCoR1/2, SWI/SNF, and NuRD complex members, as well as WD40 domain-containing proteins were unlikely to interact (Fig. 1j, Supplementary Fig. 1f, and Supplementary Data 2). Collectively, these results demonstrate that upon attenuation of Fgf signalling, Erf interacts with the NcoR1/2 transcriptional co-repressor complex (likely via Tbl1x/Tbl1xr1) in the trophoblast context (Fig. 1k).

### Erf and the NCoR1/2 complex co-occupy target regions in differentiating TSCs

**Fig. 2:**
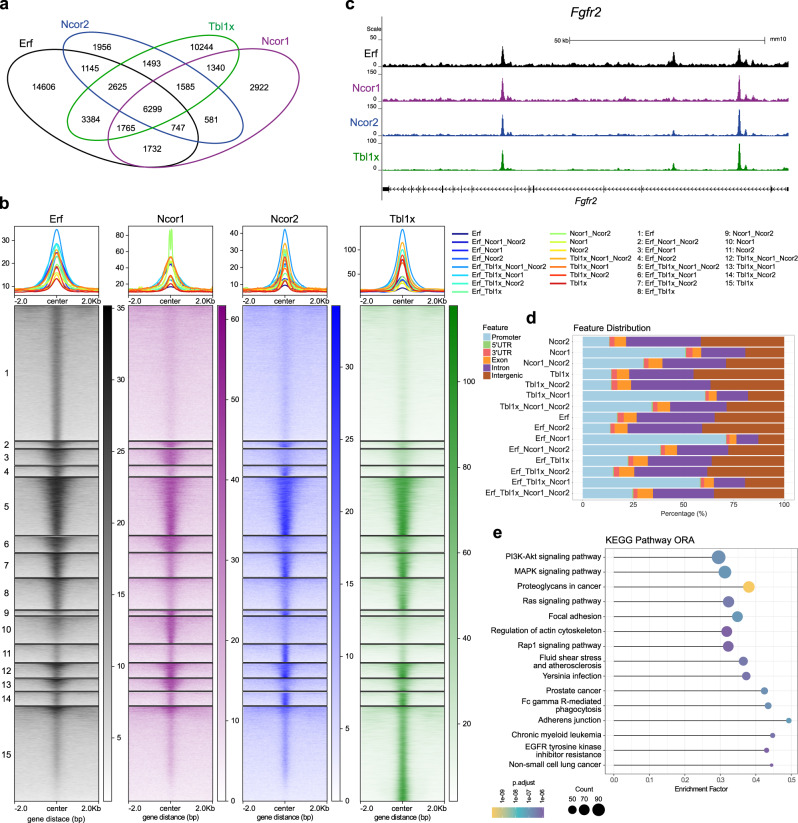
Erf and NCoR1/2 complexes co-occupy target regions. **a** Venn diagram depicting the overlap between regions bound by Erf, Ncor1, Ncor2, and Tbl1x as identified by ChIP-seq in TSCs treated for 24 h with PD. Based on IDR analysis of two biological replicates. Overlapping regions were merged. **b** Heat-map of the normalised ChIP-seq signal of Erf, Ncor1, Ncor2, and Tbl1x in regions defined by the peak overlap in (**a**). **c** Genome browser tracks of Erf, Tbl1x, Ncor1, and Ncor2 signal at the *Fgfr2* locus in TSCs after 24 h of PD0325901 treatment. **d** Feature distribution of regions occupied and co-occupied by indicated factors. **e** KEGG Pathway enrichment analysis of genes co-bound by Erf and at least one of the Ncor1, Ncor2, and Tbl1x factors. Fisher’s exact test with Benjamini–Hochberg correction was used.

The foregoing findings prompted us to hypothesise that Erf interacts with the NCoR1/2 complex, cooperatively binds to, and coregulates shared target genes. To test this model, we determined chromatin occupancy of Ncor1, Ncor2, Tbl1x and Erf in TSCs treated for 24 h with PD by chromatin immunoprecipitation followed by sequencing (ChIP-seq). We used Tbl1x as a proxy for both NCoR1 and NCoR2 sub-complexes, while Ncor1 and Ncor2 provided insights into their individual roles. To overcome antibody difficulties, the Erf endogenous locus was tagged with the V5 tag (Supplementary Fig. 2a–c). ChIP-seq with an anti-V5 antibody revealed 32303 regions bound by Erf (Fig. 2a). Importantly, motif enrichment analysis showed that a high proportion of these regions contained several known ETS DNA binding motifs as expected for Erf as a member of the ETS family of TFs, demonstrating specificity of the ChIP-seq data (Supplementary Fig. 2d). Co-localization analysis of regions bound by Ncor1, Ncor2, Tbl1x, and Erf identified 6299 (overlapping, merged) regions, co-occupied by Erf/Ncor1/Ncor2/Tbl1x (hereafter termed ETNN) (Fig. 2a). Among them were regions associated with key trophoblast genes including *Elf5*, *Fgfr2*, *Tead4*, *Hopx, Gata3*, *Tfap2c*, and *Gcm1*, indicating a potential cooperative regulation by Erf and the NCoR1/2 complex (Fig. 2a–c). In addition, we identified 1765 and 2625 regions bound by Erf/Ncor1/Tbl1x and Erf/Ncor2/Tbl1x, respectively, implying potential individual roles of Erf/NCoR1 and Erf/NCoR2 sub-complexes. As NCoR1 and NCoR2 complexes also operate outside of the Erf context, regions co-occupied by Ncor1/Ncor2/Tbl1x (1585), Ncor1/Tbl1x (1340) and Ncor2/Tbl1x (1493) were also identified. A proportion of Ncor1/2 regions devoid of Tbl1x binding may represent those bound by the related protein Tbl1xr1 (Fig. 2a, b). Feature distribution revealed that regions co-occupied by all four ETNN factors were predominantly located outside of the promoter regions, raising the possibility of enhancer regulation (Fig. 2d). Of note, we observed Ncor1 and Ncor2 binding to some ETNN targets already after 3 h of PD treatment (Supplementary Fig. 2e). Next, to gain a better understanding of processes governed by Erf/NCoR1/2, we performed the KEGG pathway enrichment analysis of the ETNN-associated genes. It demonstrated enrichment in Mapk, Pi3k/Akt, and Rap1 signalling pathways as well as in proteoglycans, adherens junctions, and focal adhesions—all playing a critical role in epithelial to mesenchymal transition, TSC identity and placental development (Fig. 2e). Taken together, our results demonstrate that Erf not only interacts with components of the NCoR1/2 complex, but also co-occupies key trophoblast genes, implying an important role of the Erf/NCoR1/2 axis in their regulation.

### Erf recruits the NCoR1/2 complex to target genes

**Fig. 3:**
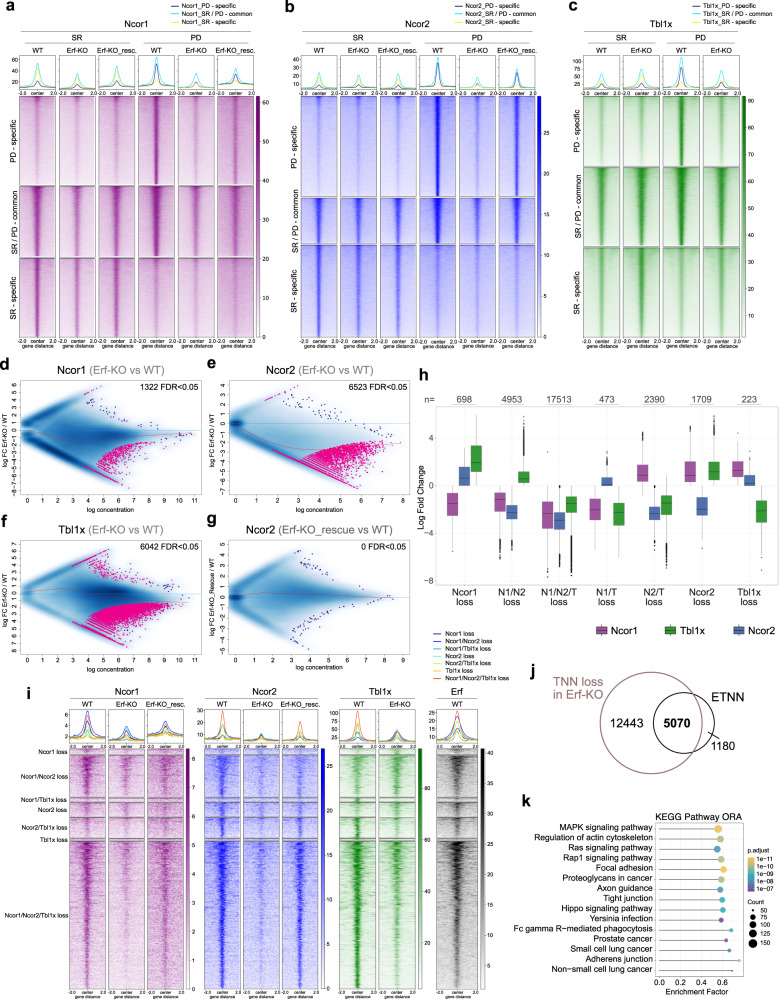
Erf recruits NCoR1/2 complexes to its target regions. **a**–**c** Heat maps of normalised Ncor1, Ncor2, and Tbl1x ChIP-seq signals in PD-specific, PD/SR-common, and SR-specific peaks for each factor, shown for WT, Erf-KO, and Erf-KO_rescue lines. **d**–**g** MA plots of differentially enriched regions for Ncor1 (**d**), Ncor2 (**e**, **g**), and Tbl1x (**f**) in Erf-KO vs WT (**d**–**f**) and Erf-KO_rescue vs WT (**g**) TSCs. Log2 fold change is plotted as a function of the log normalised ChIP-seq read counts. Regions with an FDR < 0.5 are indicated in magenta. All regions bound by any factor (based on the IDR filtered peak-sets) were included in the analysis. **h** Boxplot of the log2 fold changes of Ncor1, Ncor2, and Tbl1x signals in groups determined by combinatorial changes. Regions with FDR < 0.5 for at least one of the three factors were included. Box boundaries show the 25th to 75th percentile with the median as centre and whiskers representing the calculated maximum and minimum. Outliers are depicted by the dots. **i** Heat maps of the Ncor1, Ncor2, and Tbl1x ChIP-seq signal in regions with specific or combinatorial reduction of Ncor1, Ncor2, and/or Tbl1x binding in differentiating Erf-KO TSCs (as shown in **h**), displayed in differentiating WT, Erf-KO, and Erf-KO_rescue lines. **j** Euler diagram depicting the overlap of ETNN regions and regions with concomitant loss of signal of Ncor1, Ncor2, and Tbl1x (TNN loss) in differentiating Erf-KO cells. **k** KEGG Pathway enrichment of genes associated with TNN loss regions in differentiating Erf-KO TSCs. Fisher’s exact test with Benjamini–Hochberg correction was used.

One of the major functions of DNA-motif-binding transcriptional repressors is to recruit co-repressors, including chromatin modifying and remodelling complexes, to specific genes in a context-dependent manner. Therefore, we set out to determine whether Erf recruits the NCoR1/2 complex to its target genes. We generated Erf-KO TSCs using the CRISPR/Cas9 approach and rescue lines with the 3xFlag-Erf construct (Erf-KO_rescue) (Supplementary Fig. 3a–d). To ascertain whether the absence of Erf would affect binding patterns of NCoR1/2, we profiled the genome-wide binding of Ncor1, Ncor2, and Tbl1x in WT, Erf-KO and Erf-KO_rescue lines in SR conditions and after 24 h of PD treatment. The resulting peaks were categorised into SR-specific, SR/PD-common, and PD-specific (Fig. 3a–c). The analysis revealed numerous regions that gained (“de novo”) binding of each subunit after PD treatment (PD-specific). We also observed, on average, increased signals in the SR/PD-common regions after PD treatment. These effects were lost in the Erf-KO and largely restored in the Erf-KO_rescue lines, indicating that Erf recruits the NCoR1/2 complex in differentiating TSCs (Fig. 3a–c and Supplementary Fig. 3e, f). Corroborating our findings, differential binding analysis mainly detected regions with impaired targeting of Ncor1, Ncor2, and Tbl1x in PD-treated Erf-KO line compared to WT and the phenotype was rescued in the Erf-KO_rescue line (Fig. 3d–g and Supplementary Fig. 3g). To survey the relationships between these regions, we loosened the analysis criteria because of the known underestimation of widespread changes by the differential binding software^21,22^. Exploration of the combinatorial log fold changes of Ncor1, Ncor2 or Tbl1x identified 17513 regions with reduced binding of all three factors in PD treated Erf-KO cells (TNN loss), whereas much fewer regions specifically lost single factors (Fig. 3h–j). Of note, Ncor2 binding was most strongly lost among these factors. The TNN loss regions were enriched for genes of the MAPK signalling pathway (Fig. 3k). Importantly, they included almost all ETNN regions (81.1%, Fig. 3j) and showed robust Erf binding in differentiating WT cells (Fig. 3i). Notably, we did not observe analogous changes in occupancy of Ncor1, Ncor2, and Tbl1x in Erf-KO compared to WT TSCs cultured in SR conditions (Supplementary Fig. 3h), in line with the cytoplasmic location of Erf in self-renewing TSCs. These findings further support the direct role of Erf in the establishment of the NCoR1/2 binding patterns upon TSC differentiation and demonstrate that the absence of Erf severely affects recruitment of the NCoR1/2 complex to the shared target regions as TSCs exit self-renewal upon attenuation of Fgf/Mek signalling.

### Erf/NCoR1/2 controls expression of key trophoblast genes

**Fig. 4:**
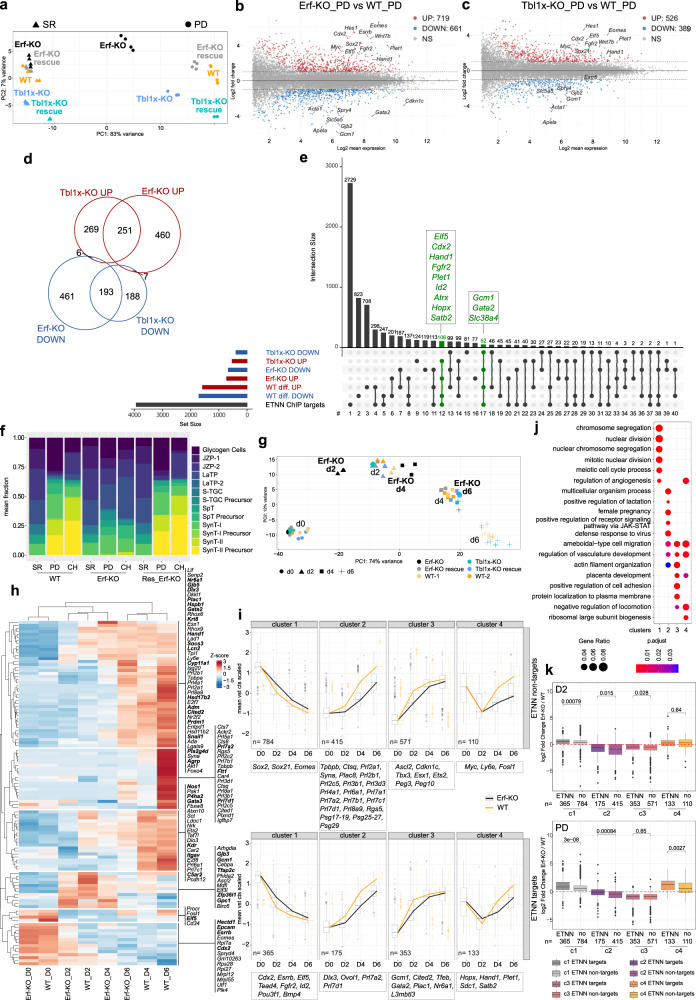
Erf/NCoR1/2 controls expression of key trophoblast genes. **a** Principal component analysis (PCA) based on global gene expression (QuantSeq) in WT, Erf-KO, Erf-KO_rescue, Tbl1x-KO, and Tbl1x-KO_rescue lines in self-renewal (SR, triangle) and after 24 h of PD0325901 treatment (PD, circle). **b** MA plot of differentially expressed genes between Erf-KO (*n* = 4 biological replicates) and WT (*n* = 4 biological replicates) lines treated for 24 h with PD. Analysis (cut-off: |log2FC | >1, p adj < 0.05, Wald-test with Benjamini–Hochberg correction) revealed 719 upregulated and 661 down-regulated genes in Erf-KO lines compared to WT. Significantly up- (UP, red) and downregulated (DOWN, blue) genes are indicated in red and blue, respectively. NS: not significant, grey. Important trophoblast-related factors are labelled. **c** MA plot of differentially expressed genes between Tbl1x-KO (*n* = 4 biological replicates) and WT (*n* = 4 biological replicates) lines treated for 24 h with PD. Analysis (cut-off: |log2FC | >1, *p* adj < 0.05, Wald-test with Benjamini–Hochberg correction) revealed 526 upregulated (UP, red) and 389 down-regulated (DOWN, blue) genes in Erf-KO lines compared to WT. NS: not significant, grey. Important trophoblast-related factors are labelled. **d** Euler diagram showing the intersection of up- and down-regulated genes (cut-off: |log2FC | >1, p adj <0.05 compared to WT) in differentiating Erf-KO and Tbl1x-KO cells. **e** Upset plot depicting the overlap between ETNN target genes, genes that were upregulated (red) and downregulated (blue) in Erf-KO and Tbl1x-KO lines after 24 h PD treatment and genes that were upregulated or down-regulated during wild-type differentiation (based on QuantSeq analysis). Groups of genes that are conversely regulated in WT and KOs are indicated as green lines in the matrix. **f** Fractions of cell type signatures found in deconvoluted bulk QuantSeq data of WT, Erf-KO, and Erf-KO_Rescue cells in SR, 24 h PD, and 48 h Fgf/CM withdrawal+CH^15^ based on a snRNAseq reference dataset from Marsh and Belloch^25^. The mean fraction (*n* = 4 biological replicates) is shown on the *y*-axis. **g** PCA based on global gene expression (QuantSeq) in WT, Erf-KO, Erf-KO_rescue, Tbl1x-KO, and Tbl1x-KO_rescue lines along a time course of Fgf/CM withdrawal (WD) for 6 days. d0: circle; d2: triangle; d4: square; d6: cross. **h** Heatmap showing the expression (Z-score) of previously identified markers^26,31,111–114^ of placental cell types in WT cells and in Erf-KO cells along the differentiation time course of Fgf/CM withdrawal for 6 days, clustered by row and column. ETNN targets marked in bold. **i** Aggregated gene expression (*n* = 3 biological replicates) in WT and Erf-KO cells during a 6-day WD differentiation time course of the 4 clusters identified by hierarchical clustering of the 3000 top variance genes (in WT WD and PD). Box boundaries show the 25th to 75th percentile with the median as centre and whiskers representing the calculated maximum and minimum. Outliers are depicted by the dots. **j** Gene ontology overrepresentation analysis of the 4 clusters (from **i**) identified by hierarchical clustering of the 3000 top variance genes. **k** Boxplots showing the log2 fold changes of the 4 clusters (from **i**) separated by ETNN and non-ETNN targets in differentiating Erf-KO compared to WT cells at d2 of WD (*n* = 3 biological replicates) and 24 h PD treatment (*n* = 4 biological replicates). *P*-values of the Wilcoxon test are indicated. Box boundaries show the 25th to 75th percentile with the median as centre and whiskers representing the calculated maximum and minimum. Outliers are depicted by the dots.

Since Erf interacts with and recruits the NCoR1/2 complex to the shared target regions, including key trophoblast regulators, we aimed to clarify which genes are bound and directly regulated by Erf/NCoR1/2. In addition to the Erf-KO and Erf-KO_rescue, we generated Tbl1x-KO and Tbl1x-KO_rescue lines using the CRISPR/Cas9 approach (Supplementary Fig. 4a–c). To determine the effects of Erf-KO and Tbl1x-KO on global gene expression patterns, we cultured WT, Erf-KO, Erf-KO_rescue, Tbl1x-KO and Tbl1x-KO_rescue TSC lines in SR conditions or treated them for 24 h with PD and performed 3' RNA sequencing (QuantSeq). The principal component analysis (PCA) showed that all the SR samples clustered away from the PD-treated samples on PC1 (Fig. 4a). Remarkably, while PD-treated WT, Erf-KO_rescue and Tbl1x-KO_rescue clustered together, the Erf-KO and Tbl1x-KO shifted towards SR samples along PC1, suggesting incomplete differentiation (Fig. 4a). Comparison between WT and Erf-KO (cut off: |logFC | >1, p adj<0.05) revealed that in SR only 15 genes showed differential expression, confirming that Erf is dispensable in self-renewing TSCs (Supplementary Fig. 4d). In stark contrast, after 24 h of PD treatment, 719 genes were upregulated and 661 down-regulated in Erf-KO compared to WT (Fig. 4b). The upregulated genes featured many self-renewal markers, including *Esrrb*, *Elf5*, *Cdx2*, *Sox21, Tead4*, and *Eomes*, while the down-regulated genes contained early differentiation markers e.g. *Gcm1* and *Cdkn1c* (Fig. 4b), in agreement with previous reports^10^. Next, we assessed differential gene expression between WT and Tbl1x-KO (cut off: |logFC | >1, p adj<0.05). In contrast to Erf-KO, the Tbl1x-KO showed gene deregulation in SR (214 upregulated and 122 downregulated), however the misregulated group included neither TSC self-renewal nor differentiation markers (Supplementary Fig. 4e). After 24 h PD treatment, we uncovered 526 upregulated and 389 downregulated genes, among them the key trophoblast regulators, including *Eomes*, *Elf5*, *Sox21, Cdx2, Gcm1*, and others (Fig. 4c). Importantly, both the Erf-KO and Tbl1x-KO differentiation phenotypes were largely rescued by ectopic expression of Erf and Tbl1x, respectively (Fig. 4a, Supplementary Fig. 4f–h). Reassuringly, we saw similar differentiation shifts after immediate Erf and Tbl1x depletion by short-hairpin RNA (shRNA) mediated knock-down (KD) (Supplementary Fig. 4i–k), confirming the same phenotypes in constitutive KO and acute depletion of Erf and Tbl1x, and excluding any adaptation effects of the Erf-KO in SR. The deregulated genes in both Erf-KO and Tbl1x-KO substantially overlapped, and their GO terms included placental development and Wnt signalling (Fig. 4d and Supplementary Fig. 4l). Uniquely regulated genes showed similar GO term enrichments in Erf-KO cells but distinct groups, like cell adhesion, in Tbl1x-KO cells (Supplementary Fig. 4l). These data suggest that Tbl1x and Erf depletion have a similar effect on gene expression in differentiating TSCs with a broader effect in Erf-KO cells. This might reflect additional functions of Tbl1x as reported^23,24^ or compensation of the phenotype by the related Tbl1xr1.

The intersection of genes bound by Erf/Ncor1/Ncor2/Tbl1x (ETNN) (Fig. 2a) with those deregulated in differentiating Erf-KO and Tbl1x-KO revealed the direct Erf/NCoR1/2 targets (groups #12, #27, #17, #31) that included key trophoblast regulators: *Cdx2*, *Elf5*, *Fgfr2*, *Atrx*, *Hopx*, *Gcm1*, and others (Fig. 4e, Supplementary Data 3 and 4). Interestingly, genes in groups #12 and #17 were inversely regulated in both KOs (Erf-KO and Tbl1x-KO) compared to WT (i.e. genes that were upregulated in Erf-KO and Tbl1x-KO were usually downregulated in WT differentiation and vice versa) (Fig. 4e). These observations suggest that disruption of Erf or Tbl1x attenuates the exit from self-renewal and delays differentiation without deviating from the normal direction of trophoblast development.

### ETNN targets are drivers of Erf-dependent differentiation

To further test the differentiation delay hypothesis, we decomposed our QuantSeq data into cell type fractions using a recently published mouse placental single-nuclei (sn)RNA-seq dataset^25^. The analysis revealed a diminished fraction of SynT-II lineage signature in Erf-KO compared to WT cells after PD treatment (Fig. 4f). These observations are in line with findings that Erf is required for the SynT-II lineage specification and the labyrinth formation during mouse placental development^10^. To explore the effects of Erf depletion on other, more differentiated trophoblast subtypes, we cultured WT and Erf-KO cells in the absence of Fgf and CM for 6 days and monitored gene expression every other day. These conditions are known to preferentially induce TGCs and SpT, and limit SynT differentiation^1^. The PCA revealed a clear shift and differentiation delay of Erf-KO compared to WT (for instance d2-WT vs d4-Erf-KO) (Fig. 4g), similar to the PD treatment. Albeit delayed, the Erf-KO did not deviate from the differentiation path lined out by the WT cells (Fig. 4g). Importantly, such delays during embryonic development are often detrimental as evidenced by the lethal placental phenotype of Erf-KO mice^10^. Decomposition of our d0-d6 withdrawal dataset based on snRNA-seq data confirmed the preferential differentiation of WT cells toward SpT and TGCs. While Erf-KO cells seemed to exhibit an ~2-day differentiation delay, the trophoblast subtype fractions remained similar (Supplementary Fig. 4m). To gain a better understanding of these events, we used previously published lineage marker sets as a reference^26^. Hierarchical clustering revealed differential expression dynamics within marker sets. For instance, established TSC markers, like *Cdx2*, *Esrrb, Eomes*, and others identified by Han et al.^26^ showed a delayed differentiation response. Transient markers, like *Gcm1*^27^ or *Cebpa*^28^ failed induction during early differentiation of Erf-KO cells as well as silencing at later phases (Fig. 4h), in line with a previous report^10^. The three biggest gene groups, despite showing distinct kinetics (in WT cells) were also homogeneously delayed in Erf-KO and included markers for different types of TGCs (e.g. members of the *Prl* and *Cts* gene families), SpT (e.g. *Ascl2*, *Tpbpa*) and glycogen cells (e.g. *Pcdh12, Gjb3*) (Fig. 4h), indicating later manifestation of an early differentiation defect. Additionally, 37 of the 112 indicated markers were ETNN targets (Fig. 4h), confirming the importance of their proper and timely regulation for placental development.

To analyse the role of ETNN targets during differentiation in an unbiased manner, we identified the 3000 top variance genes of both the PD and Fgf/CM withdrawal regimes. Over one third, 1040 top variance genes were ETNN targets (Fig. 4i and Supplementary Fig. 4n). Hierarchical clustering segregated the 3000 genes into 4 clusters that reliably reflect the regulation of the subjected genes (Fig. 4i, Supplementary Fig. 4n, and Supplementary Data 4). The upregulated gene clusters 2 and 3, enriched for the GO terms female pregnancy and positive regulation of lactation (Fig. 4i, j). Cluster 4 was transiently downregulated before expression increased during later stages of differentiation and enriched for placenta development. Cluster 1 included fast responders to attenuation of Fgf signalling, among them major regulators of TSC self-renewal (*Cdx2*, *Esrrb*, *Tead4*, and *Elf5*). Separation of clustered genes into ETNN targets and non-ETNN targets, showed a significantly greater Erf-KO-dependent deregulation among the ETNN targets in the cluster 1 (d2 and PD) and cluster 4 (PD) (Fig. 4i, k). These clusters were rapidly downregulated in WT and displayed delayed downregulation in Erf-KO cells (i.e. upregulated in Erf-KO vs WT) (Fig. 4i, k and Supplementary Fig. 4n; note the higher positive log2 fold change Erf-KO/WT in ETNN targets). This indicates that the “drivers” of the Erf-KO phenotype (i.e. self-renewal genes) reside among the ETNN targets of these early regulated clusters. Consistent with this, in cluster 2 (d2, d4, d6, and PD) and cluster 3 (d2) (i.e. differentiation genes) that were upregulated in WT and whose activation was delayed in Erf-KO, the non-ETNN targets were significantly more responsive (Fig. 4i, k and Supplementary Fig. 4n, o; note lower negative log2 fold change Erf-KO/WT in non-ETNN targets). We concluded that Erf preferentially impacts the fast-responding “first wave” of genes silenced during early TSC differentiation, and indirectly affects gene expression patterns at later stages, in line with in vivo observations^10^. Additionally, our findings demonstrate the importance of ETNN targets for trophoblast specification and as “drivers” of Erf-mediated differentiation. Overall, we showed that Erf not only recruits the NCoR1/2 complex to its target regions but also cooperatively controls a shared set of key trophoblast regulators.

### Erf/NCoR1/2 controls H3K27ac during trophoblast differentiation

**Fig. 5:**
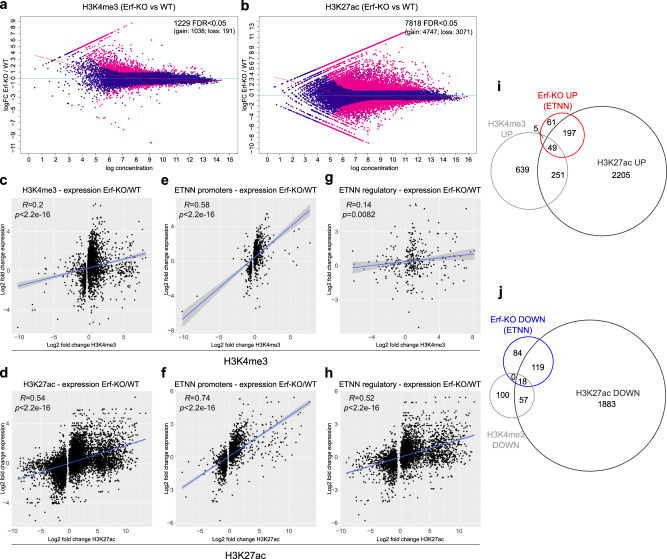
Erf depletion affects H3K27ac on target genes during differentiation. **a** MA-plot showing differentially trimethylated H3K4 on ETNN regions in differentiated Erf-KO TSCs (*n* = 2 biological replicates) compared to WT TSCs. Significant regions are indicated in pink (cut-off: |log2FC | >1, *p* adj < 0.05, Wald-test with Benjamini–Hochberg correction). **b** MA-plot showing differentially acetylated H3K27 on ETNN regions in differentiated Erf-KO TSCs (*n* = 2 biological replicates) compared to WT TSCs. Significant regions are indicated in pink (cut-off: |log2FC | >1, p adj < 0.05, Wald-test with Benjamini–Hochberg correction). **c**, **d** Correlation of differential gene expression and significant changes of H3K4me3 (**c**) and H3K27ac (**d**) in differentiated Erf-KO versus WT cells. Error bars indicate the 95% confidence limit of the linear regression model. Spearman correlation coefficient and *p*-value are indicated. **e**, **f** Correlation of differential gene expression and significant changes of H3K4me3 (**e**) and H3K27ac (**f**) on ETNN-bound promoter regions in differentiated Erf-KO versus WT cells. Error bars indicate the 95% confidence limit of the linear regression model. Spearman correlation coefficient and *p*-value are indicated. **g**, **h** Correlation of differential gene expression and significant changes of H3K4me3 (**g**) and H3K27ac (**h**) on ETNN-bound regulatory regions in differentiated Erf-KO versus WT cells. Error bars indicate the 95% confidence limit of the linear regression model. Spearman correlation coefficient and *p*-value are indicated. **i** Intersection of ETNN targets that are upregulated in Erf-KO with the respective significant changes in H3K4me3 and H3K27ac. **j** Intersection of ETNN targets that are downregulated in Erf-KO with the respective significant changes in H3K4me3 and H3K27ac.

Stem cells undergo extensive chromatin changes during differentiation. To investigate the role of Erf/NCoR1/2 in rewiring chromatin during trophoblast differentiation, we performed H3K4me3 and H3K27ac ChIP-seq in WT, Erf-KO, and Erf-KO_rescue TSCs cultured in SR and PD. We used all IDR filtered regions (see Methods) found to be modified by H3K4me3 and H3K27ac, respectively, as a reference for each differential binding analysis. We found that during WT differentiation, of 34302 H3K27ac consensus peaks 17101 were differentially regulated, while of 22135 H3K4me3 consensus peaks only 3002 (Supplementary Fig.  5a, b). These observations indicate that gene regulation at the exit from multipotency relies on highly dynamic H3K27ac, whereas H3K4me3 levels remain more stable. This is in line with previous reports that H3K27ac triggers H3K4me3 but not vice versa^29^. The overall stronger loss than gain of the two active marks during WT differentiation (H3K27ac: 9563 loss versus 7538 gain; H3K4me3: 2268 loss versus 734 gain regions), may indicate a requirement to first shut down the multipotency programme before turning on differentiation (Supplementary Fig. 5a, b). Accordingly, in differentiating Erf-KO cells we identified a strong bias towards regions with increased H3K27ac and H3K4me3 compared to WT cells (Fig. 5a, b), supporting a mostly repressive role of Erf during the exit from TSC self-renewal. These changes in histone modifications were reversed to a large extent in the Erf-KO_rescue line, confirming causality (Supplementary Fig. 5c–e). Regions that displayed increased levels of H3K27ac in differentiated Erf-KO cells exhibited increased Erf binding in WT (Supplementary Fig. 5e, f), suggesting they are the preferential Erf targets. Comparison of the changes in histone modifications and gene expression in differentiating Erf-KO cells showed a better correlation of H3K27ac changes with gene expression compared to H3K4me3 (Fig. 5c, d), despite being significant for both histone modifications. When focussing on the ETNN bound regions with histone modification changes in Erf-KO, we found that in addition to the higher number of regulatory regions bound by ETNN, H3K27ac in the promoter and particularly in the regulatory regions showed higher correlation with gene expression changes compared to H3K4me3 (Fig. 5e–h). Our observations indicate that Erf/NCoR1/2 preferentially controls gene expression via regulation of H3K27ac. These findings were further corroborated by overlapping deregulated ETNN target genes with H3K27ac and H3K4me3 changes (Fig. 5i,j). Specifically, upregulation of ETNN target genes was accompanied by H3K27ac gain in Erf-KO cells (Fig. 5i). Overall, our results demonstrated H3K27ac-dependent regulation of Erf/NCor1/2 target genes at the exit from TSC multipotency.

### Erf/NCoR1/2 regulates key enhancers during TSC differentiation

**Fig. 6:**
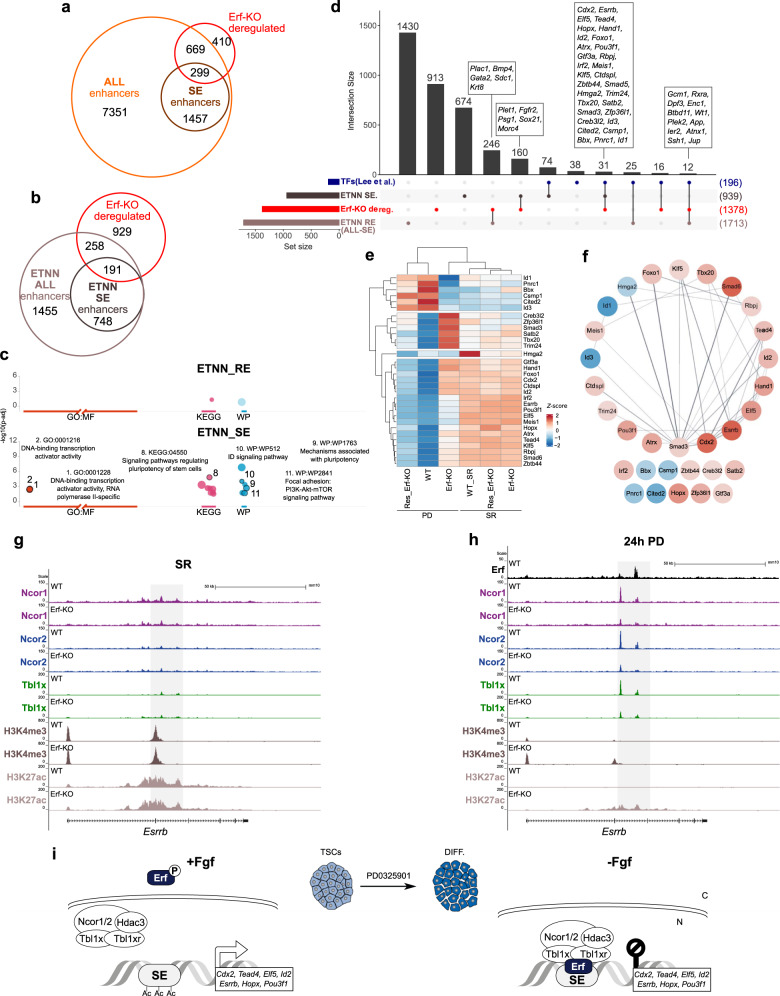
Erf/NCoR1/2 regulates key super enhancers during TSC differentiation. **a** Overlap of genes associated with enhancers (ALL_enhancers) including super enhancers (SEs) found in self-renewing and differentiating WT TSCs with genes that are deregulated in differentiating Erf-KO cells. **b** Overlap of genes associated with ETNN bound enhancers (ETNN_ALL_enhancers) including SEs (ETNN_SEs) found in self-renewing and differentiating WT TSCs with genes that are deregulated in differentiating Erf-KO cells. **c** Overrepresentation analysis of genes deregulated in differentiating Erf-KO and associated with ETNN_REs (top) and ETNN_SEs (bottom). Enrichment of GOMF, KEGG and WP database terms was measured by gProfiler2. Multiple testing was done with the internal gSCS algorithm. Only significant terms (padj ≤ 0.05) are shown. **d** Upset plot depicting intersections between genes associated with the ETNN_REs, genes associated with the ETNN_SEs, genes deregulated in differentiating Erf-KO and genes of TFs identified by Lee et al.^31^. **e** Heatmap of Z-score based on mean variance stabilised counts (*n* = 4) of the 31 ETNN_SE-associated TFs shared between this study and Lee et al.^31^ during differentiation induced by PD treatment. **f** Minimal Erf/Ncor1/2-targeted trophoblast TF network as determined by protein-protein interactions found in the STRING database. Colour scale indicates mean LFC of expression in PD treated differentiating Erf-KO compared to differentiating WT TSCs (blue, downregulated; red, upregulated, *n* = 4). **g**, **h** Genome browser tracks of Erf, Ncor1, Ncor2, Tbl1x, H3K4me3, and H3K27ac signal at the ETNN_SE-associated *Esrrb* locus in WT and Erf-KO cells in (**g**) self-renewing (SR) and (**h**) differentiating (PD) TSCs. **i** Model of Erf/NCor1/2 function in differentiating TSCs. In self-renewing TSCs, Fgf signalling leads to phosphorylation of Erf and its cytoplasmic location and expression of TSC SR marker genes. Upon abrogation of Fgf signalling, the unphosphorylated nuclear Erf recruits the NCoR1/2 complex to super enhancers (SEs) of TSC genes causing their transcriptional silencing and TSC differentiation. N:nucleus, C:cytoplasm.

Since Erf/NCoR1/2 exerts its repressive function by controlling H3K27ac, a modification commonly associated with enhancers, we sought to identify ETNN-dependent enhancers in TSCs. First, we ranked the H3K27ac regions found in SR and PD (ALL_enhancers) and categorised them into super-enhancers (SE) and regular enhancers (RE; RE = ALL_enhancers—SE) (Fig. 6a and Supplementary Fig. 6a, b) according to the ROSE algorithm. Next, the enhancer-associated genes (based on proximity mapping by ROSE) were intersected with genes misregulated in differentiating Erf-KO compared to WT. We found more RE- than SE-genes deregulated in differentiating Erf-KO cells, as expected from the sheer number of genes associated with RE (Fig. 6a). Strikingly, restriction to ETNN-bound enhancers clearly shifted the ratio of regulated genes towards the SE-associated genes (Fig. 6b, and Supplementary Data 5 and 6). To determine the functional roles of the misregulated genes associated with ETNN-bound SEs (ETNN/SE) and ETNN-bound REs (ETNN/RE) (Supplementary Data 6), we measured enrichment of functional terms in these groups (Fig. 6c). Erf-dependent ETNN/SE-associated genes significantly enriched for DNA binding transcription activator activity (GO:MF), for pathways regulating pluripotency (KEGG and WP) and for the ID pathway (WP) (Fig. 6c). This group included the master regulators of TSC identity: *Esrrb*, *Cdx2*, *Hopx*, *Elf5*, and *Tead4*. Interestingly, we also found several key regulators of the exit from naïve pluripotency in mouse embryonic stem cells (ESCs), including *Rbpj*, *Fgfr2*, and *Ctbp2* that are controlled by SEs in TSCs and upregulated in differentiating Erf-KO TSCs^30^, raising the possibility of reciprocal regulation of ESC and TSC self-renewal and differentiation.

A recent study by Lee et al. identified and partially verified a group of 196 SE-associated TFs involved in driving trophoblast stem cell identity and differentiation^31^. Since SEs represent agglomerations of “transcription factor hubs” driving master regulators of cell identity^32–34^, we used this set to filter and independently validate the Erf/NCoR1/2-dependent regulators. We intersected ETNN/SE- and ETNN/RE-associated genes with the TFs identified by Lee et al.^31^ (Lee_TFs), and with genes that were deregulated in differentiating Erf-KO cells. We found that 74 of the Lee_TFs were associated with ETNN/SEs but not responding to Erf depletion above the cut-off, indicating that they are not strictly Erf-dependent and might be regulated only in conjunction with other factors (Fig. 6d). We found an additional 160 ETNN/SE-associated genes (e.g. *Frfr2*, *Psg1*, *Plet1*, and *Sox21*) that were not included in the Lee_TFs, but misregulated in differentiating Erf-KO cells (Fig. 6d). Strikingly, the group of 31 genes that were directly bound and regulated by ETNN/SE and overlapped with the Lee_TF contained the crucial regulators of TSC identity, including *Esrrb*, *Cdx2, Tead4*, and *Elf5* (Fig. 6d). These findings indicate that several SEs in TSCs can be destabilised by the activity of a single repressor. Of note, the intersection of Erf-KO deregulated genes with the Lee_TF and the 1718 ETNN-bound REs yielded only 12 additional regulators (Fig. 6d), indicating higher relevance of ETNN/SEs. The 31 key genes formed four clusters based on gene expression in differentiating Erf-KO compared to WT cells (Fig. 6e). While cluster 1 contained genes that were not properly upregulated during differentiation (downregulated in Erf-KO vs WT), cluster 2 included genes specifically upregulated in differentiating Erf-KO cells. Cluster 3 harboured genes that were not correctly silenced upon differentiation and included the crucial TSC self-renewal factors: *Cdx2*, *Esrrb, Tead4*, and *Elf5* (Fig. 6e). The 31 TF genes were also consistently misregulated in Erf-KO cells upon Fgf/CM withdrawal (Supplementary Fig. 6c), underscoring their general relevance in trophoblast differentiation. A STRING query of these TFs identified an ETNN/SE driven gene regulatory subnetwork (GRN) at the exit from TSC multipotency (Fig. 6f). Whereas vital trophoblast functions for many of these TFs have been demonstrated in vivo and in vitro^7,35–43^, others await functional testing. Taken together, we demonstrated a central role of the Fgf/Erf/NCoR1/2 axis in decommissioning of TSC SEs at genes encoding transcriptional regulators, like *Esrrb* and *Cdx2*, and a potential but less prominent role in establishment of gene expression by targeting SEs that are activated during differentiation.

In summary, our findings support a model, where Fgf signalling sustains the TSC state by phosphorylation and cytoplasmic retention of Erf. As exemplified by the *Esrrb* locus, in SR conditions, NCoR1/2 does not bind the SE marked by both H3K27ac and H3K4me3 (Fig. 6g, see also Supplementary Fig. 6d, e). Upon inhibition of the Fgf pathway (PD), unphosphorylated nuclear Erf recruits the co-repressor complex NCoR1/2 to the SE in WT but not Erf-KO cells (Fig. 6h, see also Supplementary Fig. 6d, e). H3K27ac is lost during this transition as the SE is decommissioned, resulting in transcriptional silencing in WT but not Erf-KO cells (Fig. 6g, h). Thus, we illuminated the molecular events controlling the exit from multipotency and discovered how the Fgf/Erf/NCoR1/2 axis bridges signalling and transcription in early trophoblast cell fate decisions (Fig. 6i).

## Discussion

Determination of cell fate decisions is controlled by signalling-mediated transcriptional activation and repression; however, the molecular mechanisms of how signalling inputs are translated to transcriptional outputs remain understudied. In particular, signalling-controlled transcriptional repression is less understood, despite its critical role in exiting self-renewal and restricting lineage options in differentiating multipotent progenitors. To gain a better understanding of how Fgf/Erk signalling governs TSC identity and lineage choice, we followed the global phosphoproteomic events downstream of Mek. Using this approach, we anticipated to identify both transcriptional activators and repressors regulated by Fgf/Mek/Erk phosphorylation. While several transcriptional activators were indeed present in our dataset, including Etv4 and Etv5, their genetic ablation in mouse embryos does not display a placental phenotype^44,45^. In contrast, Erf featured as a Fgf/Mek/Erk phosphorylation target with a known repressor function and a strong placental phenotype^8,10^. These findings suggest that murine trophoblast cell identity is, at least partially, determined by an Fgf-controlled repressive mechanism. Another prominent pathway controlling transcriptional repression, is canonical WNT signalling, where in the absence of β-Catenin, the Tcf3/Tcf7l1 transcriptional repressor forms complexes with Groucho/Tle co-repressors to regulate multipotency and cell identity in diverse contexts^46,47^. Tcf7l1 represses the key naive TFs *Esrrb*, *Tfcp2l1*, *Nanog*, and *Klf5* and has recently been implicated, together with another transcriptional repressor Rbpj (a Notch pathway effector) in reconfiguring the gene regulatory network in naïve ESCs to enable their transition to the formative state and subsequent lineage commitment^48^. Interestingly all three factors (*Tcf7l1*, *Rbpj*, and *Etv5*) are regulated by SEs in TSCs. Despite all three SEs being bound by Erf/NCoR1/2, only the *Rbpj* SE is downregulated during WT differentiation and deregulated in Erf-KO TSCs in line with the combinatorial nature of SEs and cooperation of signalling pathways to drive cell fate decisions^33,49,50^. This suggests that repression plays a similar role in ESC and TSC differentiation and extends even to conversely functioning signalling targeting the same factors. Since ESCs and TSCs require reciprocal signalling cues for their self-renewal, we would speculate that certain factors exert opposite functions during their differentiation. Indeed, in TSCs with differentiation delay caused by *Erf* deletion, we found many factors upregulated, whose depletion causes a delay of ESC exit from naive pluripotency^30^. This is in line with findings in ESCs that Erf depletion rescues an exit from pluripotency phenotype caused by the MAPK pathway disruption^51^. However, our data indicate the possibility of additional direct regulation of exit from pluripotency factors by Erf complementing the recently reported Dnmt3b regulation^52^.

While transcriptional repressors usually cooperate with chromatin remodelling, modifying, and co-repressor complexes to exert transcriptional silencing, no Erf interactors have been identified previously. Here, we showed that upon attenuation of the Fgf/Erk signalling, Erf interacts with components of the co-repressor complex NCoR1/2 (Ncor1, Ncor2, Tbl1x, Tbl1xr, Hdac3, Gps2). The NCoR1/2 co-repressor complex interacts with and is recruited by TFs (e.g. NFkb1, ETV6, BCL-6, c-Jun, Foxp1) and ligand-dependent nuclear receptors (e.g. thyroid hormone and retinoic acid receptors) in a sequence-specific manner to transcriptionally silence target genes^53,54^. Although the Erf/NCoR1/2 interaction has not been reported before, other Ets factors, including ETS1, ETS2 and ETV6 are known NCoR1/2 interactors in other contexts^55,56^. Additionally, upon reanalysis of published ChIP-seq datasets together with our data, we found several factors that show a correlative binding pattern with NCoR1/2 (Supplementary Fig. 7a). These factors, including Sox2 and Elf5, are potential NCoR1/2 collaborators in self-renewal and could be replaced by Erf, providing a molecular switch during the TSC exit from multipotency. NCoR1/2 was not found to interact with neither Elf5^43^ nor Sox2 in SR TSCs (Supplementary Fig. 7b); however, the lack of a direct interaction does not exclude cooperative regulation of target genes. On the other hand, protein interactions do not necessarily imply recruitment and cooperation at target genes. Integration of the genomic binding patterns of Erf/NCoR1/2 subunits and transcriptomics data of Erf-KO cells demonstrated Erf-dependent recruitment of NCoR1/2 and regulation of its targets.

Genetic ablation of *Erf* results in a differentiation block of chorionic trophoblast, lack of chorioallantoic fusion and embryonic lethality at E10.5. Mouse TSCs derived from these KO embryos show a differentiation defect and a delay in specification^10^. Consistently, our Erf-KO and Erf-KD lines exhibited persistent expression of self-renewal markers and failed to induce the differentiation marker *Gcm1* upon a 24 h PD treatment. Similarly, withdrawal of Fgf and CM for 6 days also resulted in an Erf-KO differentiation delay, affecting later lineage-specific (e.g., TGCs and SpT) markers. This suggests that delaying the exit from multipotency as a very early event of trophoblast development leads to severe defects in subsequent lineage progression in vitro and embryo development in vivo. Strikingly, deletion of the NCoR1/2 component *Tbl1x* resulted in a highly similar phenotype, corroborated by a massive overlap of misregulated genes between Erf-KO/-KD and Tbl1x-KO/-KD, including key trophoblast TFs driven by Fgf signalling^7^ and *Gcm1*. Importantly, a high proportion of these misregulated genes were bound by both Erf and NCoR1/2 components, confirming their direct regulation.

NCoR1/2 has been implicated in transcriptional regulation of cell fate decisions in diverse cellular contexts, including ESC differentiation, haematopoiesis, adipogenesis but also in lungs and muscles^57,58^. During murine embryonic development Ncor1 and Ncor2 are critical for development of the CNS, maintenance and differentiation of neural stem cells^59–62^, haematopoiesis and heart development, and ablation of these genes results in embryonic lethality at E14.5 and E16.6, respectively^59,60^. Importantly, flawed NCoR1/2 activity has been linked to various diseases, including several types of leukaemia and other cancers^63^. Thus, our reported control of trophoblast cell fates by the Erf-driven recruitment of NCoR1/2 to target genes and their silencing is consistent with the function of NCoR1/2 as a transcriptional regulator of cell identity. In a broader context, several other co-repressor and chromatin remodelling complexes, including NuRD, Sin3a, and SWI/SNF, have been demonstrated to govern self-renewal and differentiation^64–66^.

While NCoR1/2 predominantly exerts transcriptional silencing, it has also been involved in transcriptional activation^24,57^. Indeed, our analysis revealed a subset of Erf/NCoR1/2 target genes that were down-regulated in Erf-KO and Tbl1x-KO, suggesting that Erf/NCoR1/2 may drive their activation during TSC differentiation. Among them was the SynT-II master regulator *Gcm1* that requires Wnt/β-Catenin signalling for its activation. Interestingly, the activator roles of NCoR1/2 components Tbl1x/Tbl1xr1 have been linked to Wnt signalling, and they have been shown to interact with β-Catenin and activate transcription^23,67^. Importantly, Wnt7b-KO, Rspo3-KO (ligands of the Wnt pathway), Gcm1-KO, and Erf-KO mice exhibit highly similar placental phenotypes resulting in embryonic lethality by E10.5^10,68–70^ and Erf-KO and Tbl1x-KO showed similar TSC differentiation defects. Taken these findings together, it is tempting to speculate that the Erf/NCoR1/2 could act as a molecular effector of Fgf/Wnt signalling that drives chorioallantoic fusion during placental development. Specifically, upon abrogation of Fgf signalling, nuclear Erf would recruit the NCoR1/2 complex to the *Gcm1* promoter. Next, upon activation of the Wnt pathway, Tbl1x/Tbl1xr would cooperate with β-Catenin to drive *Gcm1* transcription. Consistent with this model, in vitro SynT-II differentiation requires activation of Wnt signalling in the absence of Fgf^15^. However, additional experiments are required to test this hypothesis.

Since we postulate that Erf together with the NCoR1/2 complex cooperatively regulates trophoblast development, and since Erf-KO impairs it, it would be expected that ablation of NCoR1/2 would also result in trophoblast defects. The lack of a reported placental phenotype in single Ncor1- and Ncor2-KO may result from compensatory mechanisms due to their high similarity. The Ncor1/Ncor2-dKO and the Hdac3-KO embryos are lethal before the placenta is formed, while the Tbl1xr1-KO and Tbl1x-KO mice have not been analysed. Thus, the role of NCoR1/2 in trophoblast development in vivo remains to be fully determined. Importantly, here we showed that genetic ablation of Tbl1x in TSCs resulted in differentiation defect and the phenotype was highly similar to the Erf-KO, supporting the functional Erf/NCoR1/2 link. It will be interesting to test whether the Erf/NCoR1/2 interaction operates in developmental contexts other than trophoblast. Observations that ablation of Ncor1 or Erf in the embryo proper resulted in a shared phenotype involving impaired haematopoiesis, severe anaemia and lethality around E14.5^59,71^, indicate that the Erf/NCoR1/2 axis may play a more general role.

Similar to other stem cell systems^33,34,72,73^, TSCs harbour SEs associated with TFs vital for development^31^. How and if such trophoblast SEs are regulated by signalling pathways remained elusive. We observed global H3K27ac redistribution upon TSC exit from self-renewal, prompting us to determine REs and SEs based on H3K27ac. The SE-associated genes were enriched in TFs, among them the key regulators of TSC self-renewal including *Elf5*, *Tead4*, *Cdx2*, *Esrrb*, *Tfap2c*, and *Tead1*. Importantly, a high proportion of these SEs was targeted by the Erf/NCoR1/2 complex in differentiating TSCs and associated with genes misregulated in the Erf-KO. This is in line with the recent finding that NCoR1/2 antagonises enhancer activation by modulating/removing H3K27ac in macrophages and during adipogenesis^12,74^ and could be translated in a role of Erf/NCoR1/2 in actively shutting down a TF subset of the TSC GRN. In TSCs, Erf-mediated recruitment of NCoR1/2 seems to be a crucial step to initiate enhancer decommissioning. Many ETNN/SE-associated genes were not misregulated upon Erf depletion, owing to their combinatorial nature and indicating that the regulation of only a subset of ETNN/SEs was strictly Erf/NCoR1/2-dependent at the exit from TSC multipotency. However, this subset included several master regulator genes, such as *Cdx2* and *Esrrb*, which lose differentiation cues in Erf-KO TSCs as there were no signs of enhancer decommissioning or downregulation upon PD treatment. Consistently, *Cdx2* overexpression induces conversion of ESCs into TSCs and its retained expression fortifies the self-renewal and polar characteristics of TSCs^74,75^. *Esrrb* has been shown together with *Sox2*, to render TSC self-renewal Fgf-independent when overexpressed^76^. The expression of other factors that are implicated in lineage specific differentiation were even upregulated above WT self-renewal levels in differentiating Erf-depleted TSCs. Interestingly, *Hand1*, *Satb2*, *Creb3l2*, *Trim24*, and *Smad3* were shown to be downregulated during the very early phase of TSC differentiation^31^ (Fig. 6e and Supplementary Fig.  6c). This indicates a role of these factors in TSC self-renewal and the necessity of a complete exit from multipotency to gain competence for differentiation and responsiveness to developmental cues before lineage choice in the placenta, similar to ESCs^77^. Differentiation-specific enhancers showing attenuated H3K27ac deposition in differentiating Erf-KO TSCs, indicated involvement of Erf/NCoR1/2 in activation or release of repressive mechanisms on key regulators of differentiation. Further experiments are needed to complete our view on these activated enhancers.

Taken together, we propose a model where Fgf sequentially controls TSC self-renewal and differentiation by direct transcriptional activation and repression mechanisms, respectively. This model is also consistent with our observations that Erf-KO and Tbl1x-KO TSCs exhibit a delayed exit from self-renewal. Thus, we uncovered a highly dynamic Fgf/Erf/NCoR1/2 axis that integrates signalling cues and the downstream molecular effectors at regulatory elements of master regulators of trophoblast identity (Fig. 6i), governing trophoblast cell fate decisions during murine placental development.

## Methods

### Cell culture

Mouse TS cells (TS EGFP line, a kind gift of Prof. Janet Rossant, University of Toronto, Canada) were cultured as described previously^78^. Briefly, TS cells were grown in a standard TS medium (RPMI 1640 supplemented with 20% foetal calf serum, 2 mM L-Glutamine, 2 mM sodium pyruvate and 100 mM 2-mercaptoethanol) containing 70% mouse embryonic fibroblast -conditioned medium (CM) and 100 ng/ml Fgf2 and 1 µg/ml heparin. Cells were split every 3^rd^ day using trypsin. Transfections were performed for 6 h in OptiMEM media supplemented with Fgf2 and heparin using 1% Lipofectamine 3000 (Life Technologies) on nonadherent dishes. After 24 h, cells were selected with 300 μg/ml G418 (A1720, Sigma). To induce differentiation, TSCs were either exposed to 3 µM PD0325901 (Tocris) in full TSC media for the indicated length of time (usually 24 h) or cultured in the TS basal media (RPMI 1640 supplemented with 20% foetal calf serum, 2mM L-Glutamine, 2 mM sodium pyruvate, 100 mM 2-mercaptoethanol, no heparin, no Fgf2, no CM) for the indicated length of time (usually 2, 4, or 6 days).

### Genetic manipulations

To generate Tbl1x-KO 12 µg Tbl1x_g1 and 12 µg of Tbl1x_g2 gRNAs were mixed with 5 µg of the recombinant Cas9 protein in a cleavage buffer (200 mM Hepes pH7.5, 1.5 M KCl, 5 mM DTT, 1 mM EDTA) and incubated for 5 min at RT. The Cas9/Ribonucleoproteins were electroporated into 1 × 10^6^ TS-GFP cells with 1400 V and three pulses of 10 ms width using the Neon^TM^ transfection system. Single clones were picked and genotyped. Generation of CRISPR/Cas9 Tbl1x-KO was performed in collaboration with the VBCF Protein Technologies Facility. To generate the Erf-KO constructs, we cloned the Erf_g1 and Erf_g2 gRNAs into the *BbsI* site of the pX459 vector. 2 × 10^5^ TS-GFP cells were transfected with 1.5ug of each construct using the Neon^TM^ transfection system. Following 400 µg/ml G418 selection, single clones were picked and genotyped. The Erf-KO_Erf and Tbl1x-KO_Tbl1x rescue lines were generated by transfection of the Erf-KO and Tbl1x-KO lines with the PiggyBac-CAG-Avi-Erf-3xFlag-ires-Neo and PiggyBac-CAG-Avi-Tbl1x-3xFlag-ires-Neo constructs, respectively, and transposase, followed by selection with 350 µg/ml G418. The endogenously tagged Erf-V5 line was generated using tracrRNA, crRNA, and ssODN (Integrated DNA Technologies) as described^79^. For details see Supplementary Table 1. Erf phospho-mutants were generated using primers listed in Supplementary Table 1 and carried following mutations: M5 (S21A, S185A, S190A, S534A, S327A); M6 (S161A, T529A, S246A, S251A, T357A, T148A). M5, M6 and WT Erf were cloned into a doxycycline (dox) inducible PiggyBac-Tre-Dest-rTA-HSV-neo vector (a kind gift from Dr. Joerg Betschinger, Friedrich Miescher Institute for Biomedical Research, Basel, Switzerland). Erf-KO TSGFP cells were transfected with each construct, selected with 300 μg/ml G418, and expression was induced with 1 μg/ml dox for 24 h. To knock down Erf and Tbl1x, we generated pLKO.1-based constructs containing shRNA against Erf (TRCN0000109356 and TRCN0000109359), Tbl1x (TRCN0000084155 and TRCN0000084156), and an shRNA against GFP as a control by cloning annealed oligos into the AgeI/EcoRI sites of pLKO.1-neo (13425, Addgene). The plasmids were simultaneously transfected into HEK293T cells: pLKO.1 (individual constructs containing different shRNA), psPAX2 (encodes Gag and Pol sequences to package the lentivirus), and pMD2.G (encodes the G protein envelope protein) using lipofectamine 3000 (Thermo Fisher Scientific). Supernatant was collected after 48 h and used to transduce TSC for a minimum of 16 h. Cells were selected using 350 μg/ml G418 (A1720, Sigma-Aldrich). To generate Erf- and Tbl1x expression/rescue constructs, Erf- and Tbl1x-coding sequences were cloned to result in PiggyBac-CAG-Avi-Erf-3xFlag-ires-Neo and PiggyBac-CAG-Avi-Tbl1x-3xFlag-ires-Neo constructs, respectively. TSGFP cells were transfected with each construct along with the empty vector control using Lipofectamine 3000 (Invitrogen) and selected with G418 350 μg/ml G418 (A1720, Sigma).

### Chromatin immunoprecipitation

Chromatin immunoprecipitations were carried out as described^7,30^. Erf-V5 and WT TSC lines (1–2 × 10^8^ cells, each) were fixed in 2 mM Di(N-succinimidyl) glutarate (DSG) (80424, Sigma) in PBS at room temperature (RT) for 45 min. After washing with PBS, cells were fixed again in 1% formaldehyde (28908, Sigma) in TS base media at RT for 12 min. Fixation was stopped by adding glycine to a final concentration of 0.125 M. Cells were washed twice with PBS and resuspended in wash buffer 1 (10 mM Hepes pH7.5, 10 mM EDTA, 0.5 mM EGTA, and 0.75% Triton X-100) and incubated at 4 C for 10 min. After pelleting, cells were resuspended in wash buffer 2 (10 mM Hepes pH7.5, 200 mM NaCl, 1 mM EDTA and 0.5 mM EGTA) and incubated at 4 °C for 10 min. After pelleting, cells were lysed in the lysis/sonication buffer (150 mM NaCl, 25 mM Tris pH 7.5, 5 mM EDTA, 0.1%Triton, 1% SDS and 0.5% sodium deoxycholate) with cOmplete^TM^ EDTA-free Protease Inhibitor Cocktail (11873580001, Sigma) on ice for 30 min. Chromatin was sonicated 15 s on /30 s off for 23-25 cycles using the UW2070 sonicator (Bandelin) to the average 300-bp fragments. Chromatin was diluted 1/10 with the dilution buffer (150 mM NaCl, 25 mM Tris pH 7.5, 5 mM EDTA, 1% Triton X-100, 0.1% SDS and 0.5 % sodium deoxycholate) containing complete protease inhibitors. IP was performed with pre-washed anti-V5 Agarose Affinity Gel (Sigma-Aldrich, A7345) overnight at 4 °C with rotation. Beads were washed at 4 °C with buffer A three times, buffer B (50 mM Tris pH 8.0, 500 mM NaCl, 0.1% SDS, 0.5 % sodium deoxycholate, 1% NP-40 and 1 mM EDTA), buffer C (50 mM Tris pH 8.0, 250 mM LiCl, 0.5% sodium deoxycholate, 1% NP-40 and 1 mM EDTA) and rinsed with TE buffer. DNA was eluted from beads in the elution buffer. Samples were treated with RNAse A and Proteinase K and reverse-crosslinked overnight by incubation for 2 h at 37 °C, followed by 12 h at 65 C. DNA was purified on the PCR purification columns (Qiagen). To generate a library, DNA from 2-4 IPs was pooled and the NEB Next Ultra II DNA library preparation master mix (New England Biolabs, E7645) was used according to the manufacturer’s instructions. Libraries were sequenced on an Illumina HiSeq 2500 sequencer in 100-base and 50-base pair (bp) single-end mode. For Tbl1x, Ncor1, Ncor2, H3K27ac, and H3K4me3 ChIP-seq on WT and Erf-KO TSC lines, cells were fixed in 1% FA for 10 min and fixation was quenched by 0.125 M glycine at RT. Cells were scraped in PBS, centrifuged at 500 g for 5 min and pellet was resuspended in an ice-cold IP-buffer, a 2:1 mixture of SDS-buffer (100 mM NaCl, 50 mM Tris-Cl pH 8.0, 5 mM EDTA pH 8.0, 0.5% SDS) and Triton-X-buffer (100 mM NaCl, 100 mM Tris-Cl pH 8.6, 5 mM EDTA pH 8.0, 5% Triton X), containing complete protease inhibitor tabs (11873580001, Sigma). After 30 min incubation on ice chromatin was sonicated 15 s on (pulsed, with 90% actual sonication time) /30 s off for 20 cycles using the UW2070 sonicator (Bandelin) at ~40% power. 30 µg and 10 µg chromatin (DNA equivalent) were diluted to 500 µl and precipitated at 4 °C overnight using the following antibodies: anti-Ncor1 (Abcam, ab3482), anti-Ncor2 (Abcam, ab5802), anti-Tbl1x (Proteintech, 13540-1-AP), anti-H3K27ac (Diagenode, C15410196), anti-H3K4me3 (Diagenode, C15410003). For ChIP of histone modifications, we added 0.8 µg Spike-In antibody (61686, Active Motif) and 25 ng Spike-In chromatin per ChIP (53083, Active Motif). 50 µl Dynabeads Protein G (10004D, Invitrogen) equilibrated in IP-buffer, blocked with 1 mg/ml BSA and tRNA at 4 °C for 1 h and washed with IP-buffer were added to the IP reaction and incubated for 3 h at 4 °C. ChIPs were washed three times with Low Salt Buffer (50 mM HEPES pH 7.5, 140 mM NaCl, 1% Triton X), once with High Salt Buffer (50 mM HEPES pH 7.5, 500 mM NaCl, 1% Triton X) and once with TE buffer before elution with 200 µl Elution Buffer (1% SDS, 100 mM NaHCO_3_) per reaction. Eluate was separated from the beads and decrosslinked over night at 65 °C. After RNaseA and proteinase K treatment 5 ng of DNA were subjected to library preparation (see above). Libraries were sequenced paired-end 50 bp on an Illumina NovaSeq 6000 SP1 flowcell.

### QuantSeq

RNA was extracted using the RNeasy mini kit (Qiagen) and treated with DNaseI (Qiagen). Indexed libraries were prepared with 500 ng or 250 ng RNA using QuantSEQ 3' mRNA-Seq Library Prep Kit FWD for Illumina (015.96, Lexogen) according to manufacturer’s recommendations. The barcoded libraries were pooled and sequenced with a 100-base-pair single-read and 50-base-pair single-read protocol on an Illumina HiSeq 2500 sequencer.

### Co-immunoprecipitation/western blotting

Cells were lysed using Hunt-Buffer (20 mM Tris-HCl pH 8, 100 mM NaCl, 1 mM EDTA, 0,5% NP-40) with a protease inhibitor (Roche). To 1500 µg of protein 7 µg of the antibody (anti-Tbl1x, 13540-1-AP, Proteintech and anti-IgG, NI0, Sigma-Aldrich) was added and incubated overnight at 4 °C with rotation. Next, 50 µl of pre-washed and pre-blocked (1 µg/µl BSA) magnetic protein G Dynabeads (Thermo Fischer Scientific, 10765583) were added and incubated at 4 °C on a rotor at 20 rpm for 3 h. Samples were washed 3x 15 min with rotation using Hunt-Buffer/protease inhibitor and eluted from beads in 30 µl of 2x Laemmli buffer and used for western blotting with the following antibodies: anti-V5 (Sigma-Aldrich, V8012), anti-Tbl1x (Proteintech, 13540-1-AP), anti-Ncor1 (Abcam, ab3482), anti-Ncor2 (Abcam, ab5802), anti-Hdac3 antibody (Abcam, ab7030), and anti-Sox2 (R&D, AF2018).

### Western blotting

Whole cell lysates were prepared with TG buffer 20 mM Tris-HCl pH 7.5, 137 mM NaCl, 1 mM EGTA, 1% Triton X-100, 10% glycerol and 1.5 mM MgCl_2_. Following primary antibodies were used: anti-Flag (Sigma-Aldrich, F1804), anti-V5 (Sigma-Aldrich, V8012), anti-Tbl1x (Proteintech, 13540-1-AP), anti-Erf (Santa Cruz, sc-398269), anti-Tubulin (Abcam, ab6160), anti-Erk1/2 (Cell Signalling, 4695), anti-phosphoErk1/2 (Cell Signalling, 9106), anti-LaminB1 (Santa Cruz, sc-374015). Following secondary antibodies were used: anti-rabbit IgG(L)-HRPP (Dianova, 211-032-171) and anti-mouse IgG(L)-HRP (Dianova, 115-035-174); note both antibodies detect light but not heavy chains.

### Mass spectrometry analysis

Phosphoproteome: TSGFP cells were treated with 3 µM PD0325901 in standard TS media for 0, 1, 5, 15 and 30 min, lysed for 15 min at room temperature in a lysis buffer (8 M Urea, 20 mM Hepes, 15 mM DTT) supplemented with phosphatase inhibitor cocktail 2 and 3, (Sigma-Aldrich), and sonicated (20 cycles: 10 s ON, 30 s OFF). The lysates containing 1.5 mg of protein in 1 ml were supplemented with 250 units of Benzonase (250 U/µl, purity grade I, Merck) and incubated at 37 °C for 1 h. The alkylation was performed by adding 30 mM Iodoacetamide and incubating for 30 min in the dark. The reaction was quenched by addition of 7.5 mM DTT and incubation at room temperature for 30 min. The samples were diluted to 6 M urea by addition of 100 mM Ammoniumhydrogencarbonate (ABC) and the proteins were digested with Lys-C at an enzyme to protein ratio of 1:50 for 2 h at 37 °C. Subsequently, the samples were further diluted to 4 M urea with 100 mM ABC, and trypsin was added in a ratio of 1:75. After incubation for 2 h at 37 °C, the samples were further diluted to 2 M, trypsin was added another time in a ratio of 1:75 and the incubation at 37 °C was continued overnight. The samples were acidified by addition of Trifluoroacetic acid (TFA) to reach pH 2. Peptides were desalted using C18 cartridges (Sep-Pak Vac 3cc (200 mg), Waters). Peptides were eluted with 70% Acetonitrile (ACN) and 0.1% Formic Acid (FA), followed by freeze-drying. TMT labelling: the lyophilised peptides were dissolved in 240 μl of 100 mM Triethylammonium bicarbonate. TMT10-plex labelling reagents (Three vials per label) were dissolved in 41 μl of anhydrous acetonitrile. Three vials per label (2.4 mg of reagent) were added to the peptide samples (1.5 mg peptide per sample) and incubated for 1 h. After quenching with 5% hydroxylamine, the samples were mixed in several steps to achieve a correct mixing ratio of 1:1:1:1:1:1:1:1:1:1. (median of Top 500 proteins). Following labelling, peptides were again freeze-dried, dissolved in TFA 0.1%, and desalted using C18 cartridges (Sep-Pak Vac 6cc-500 mg, Waters). Peptides were eluted with 70% ACN and 0.1% FA, followed by freeze-drying. Phosphopeptide enrichment and fractionation: phosphopeptides were enriched using TiO_2_ by first dissolving the dried peptide mixture in 4 ml of TiO_2_ loading buffer (300 mg/ml lactic acid (Sigma-Aldrich), 80% ACN, 0.1% TFA) and incubating them with 120 mg of TiO_2_ resin (Titansphere bulk media, 5 micron, GL Science) for 60 min at room temperature with over end rotation. For washing and elution the bead suspension was split and transferred to 8 Mobicol (MoBiTec) spin columns with a 10 µm pore filter at the outlet. The TiO_2_ beads in each spin column were washed with 8 × 500 µl of TiO_2_loading buffer, 8 × 500 µl of 80% ACN, 0.1% TFA, 4 × 500 µl of 1% ACN, 0.1% TFA, 4 × 500 µl of 1% ACN, 0.1% FA. Bound peptides were eluted from the resin by addition of 3 × 200 µl 0.3 M NH_4_OH, 2 × 200 µl 0.7 M NH_4_OH and 2 × 150 µl 0.4 M NH_4_OH and 40% ACN, eluates were unified and freeze-dried. The lyophilised peptide sample was dissolved in SCX buffer A (5 mM phosphate buffer pH 2.7, 15% ACN). The SCX fractionation was performed on an Ultimate system (Thermo Fisher Scientific) using a TSKgel SP-25W (ToSOH) column (5 μm particles, 1 mm i.d. x 300 mm) at a flow rate of 35 μl/min. For the separation, a ternary gradient was used, starting with 100% buffer A for 10 min, followed by a linear increase to 10% buffer B (5 mM phosphate buffer pH 2.7, 1 M NaCl, 15% ACN) and 50% buffer C (5 mM phosphate buffer pH 6.0, 15% ACN) in 60 min, to 25% buffer B and 50% buffer C in next 10 min, to 50% buffer B and 50% buffer C in next 5 min and an isocratic elution for further 15 min. The flow-through was collected as a single fraction, along the gradient fractions were collected every minute and stored before analysis.

APMS experiment: Erf- and Tbl1x-coding sequences were cloned to result in PiggyBac-CAG-Avi-Erf-3xFlag-ires-Neo and PiggyBac-CAG-Avi-Tbl1x-3xFlag-ires-Neo constructs, respectively. TSGFP cells were transfected with each construct along with the empty vector control using Lipofectamine 3000 (Invitrogen), selected with 350 μg/ml G418 and expanded in 10 15-cm dishes. Co-immunoprecipitation was performed as described before^7^. Cells were washed in PBS, harvested, resuspended in Buffer A (10 mM Hepes pH 7.6, 1.5 mM MgCl2, and 10 mM KCl) and disrupted by 10 strokes in a dounce homogenizer. Extracts were spun down and the pellet resuspended in Buffer C (20 mM Hepes pH 7.6, 25% Glycerol, 420 mM NaCl, 1.5 mM MgCl2, and 0.2 mM EDTA), passed through a 19-G needle and dialysed to Buffer D (20 mM Hepes pH 7.6, 20% Glycerol, 100 mM KCl, 1.5 mM MgCl2, and 0.2 mM EDTA) using dialysis cassettes (Fisher Scientific). Anti-FLAG M2 agarose beads (Sigma-Aldrich, A2220) equilibrated in buffer D were added to 1.5 ml of nuclear extract in No Stick microcentrifuge tubes (Alpha Laboratories) and incubated for 3 h at 4 C in the presence of Benzonase (Novagen). Beads were washed five times for 5 min with buffer D containing 0.02%NP-40 (C-100*) and six times with (20 mM Hepes, 10% Glycerol, 100 mM KCl, 1.5 mM MgCl_2_). Next, the beads were resuspended in 60ul of 100 mM ammonium bicarbonate (ABC), supplemented with 400 ng of lysyl endopeptidase (Lys-C, Fujifilm Wako Pure Chemical Corporation) and incubated for 4 h on a Thermo-shaker with 1200 rpm at 37 °C. The supernatant was transferred to a fresh tube and reduced with 0.5 mM Tris 2-carboxyethyl phosphine hydrochloride (TCEP, Sigma) for 30 min at 60 °C and alkylated in 3 mM methyl methanethiosulfonate (MMTS, Fluka) for 30 min at room temperature. Subsequently, the sample was digested with 400 ng trypsin (Trypsin Gold, Promega) at 37 °C overnight. The digest was acidified by addition of trifluoroacetic acid (TFA, Pierce) to 1%. A similar aliquot of each sample (30%) was analysed by LC-MS/MS.

HPLC-MS: the system used was an UltiMate 3000 RSLC nano system coupled to a Q Exactive HF or an Orbitrap Exploris 480 mass spectrometer, equipped with a Proxeon nanospray source (all from Thermo Fisher Scientific). Peptides were loaded onto a trap column (Thermo Fisher Scientific, PepMap C18, 5 mm × 300 μm ID, 5 μm particles, 100 Å pore size) at a flow rate of 25 μL/min using 0.1% TFA as mobile phase. After 10 min, the trap column was switched in line with the analytical column (Thermo Fisher Scientific, PepMap C18, 500 mm × 75 μm ID, 2 μm, 100 Å). Peptides were eluted using a flow rate of 230 nl/min, starting with the mobile phases 98% A (0.1% formic acid in water) and 2% B (80% acetonitrile, 0.1% formic acid) and linearly increasing to 35% B over the next 60 min (TMT SCX fractions) or 180 min (APMS experiments), followed by a gradient to 90% B in 5 min, staying there for 5 min and decreasing in 2 min back to the gradient 98% A and 2% B for equilibration at 30 °C. The Q Exactive HF mass spectrometer was operated in data-dependent mode, using a full scan (m/z range 350–1650, resolution of 120 000, target value 3E6 for TMT fractions, m/z range 380–1500, resolution of 60 000, target value 1E6 for APMS) followed by MS/MS scans (resolution of 60000, target value 1E5, maximum injection time 250 ms for TMT fractions, resolution of 30000, target value 1E5, maximum injection time 105 ms for APMS) of the 10 most abundant ions. MS/MS spectra were acquired using normalised collision energy 35% (TMT) and 27% (APMS) and an isolation width of 1.2 m/z (TMT) and 1.4 m/z (APMS). For the detection of the TMT reporter ions a fixed first mass of 115 m/z was set for the MS/MS scans. Precursor ions selected for fragmentation were put on a dynamic exclusion list for 30 s (TMT) and 60 s (APMS). The intensity threshold was set to 4E4. The Orbitrap Exploris 480 mass spectrometer was operated in data-dependent mode, performing a full scan (m/z range 350-1200, resolution 60,000, normalized AGC target 100%) at 3 different compensation voltages (CV −45, −60, −75), followed each by MS/MS scans of the most abundant ions for a cycle time of 0.9 s per CV. MS/MS spectra were acquired using HCD collision energy of 30, isolation width of 1.0 m/z, orbitrap resolution of 30.000, normalized AGC target 200%, minimum intensity of 25.000 and maximum injection time of 100 ms. Precursor ions selected for fragmentation (include charge state 2–6) were excluded for 45 s. The monoisotopic precursor selection (MIPS) filter and exclude isotopes feature were enabled.

### Immunostaining

Cells were fixed in 4% paraformaldehyde/PBS for 20 min at 4 °C, permeabilised and blocked for 30 min in 4% donkey serum and 0.1% Triton X-100/PBS. The following primary antibodies were used: anti-Flag (F1804, Sigma-Aldrich), anti-V5 (Sigma-Aldrich, V8012). Alexa Fluor-conjugated secondary antibodies (Invitrogen) were applied at 1:1000 in 4% donkey serum and 0.1% Tween-20 in PBS blocking solution. Cells were counterstained with 4,6-diamidino-2-phenylindole (DAPI) and imaged using Zeiss Imager A2 confocal microscope with the Zen2012 software.

### RT-QPCR

RNA was extracted using the RNeasy mini kit (Qiagen) and treated with DNaseI (Qiagen) according to the manufacturer’s protocol. cDNA was synthesised using 1.5–3 µg RNA primed with random hexamers according to the RevertAid Reverse Transcriptase protocol (Thermo Scientific EP0442). DNA was diluted and qPCR performed using GoTaq qPCR Master Mix (A6002, Promega). Results are shown as means of indicated number of biological replicates (*n*) +/− S.E.M. Statistical significance was determined using a two-tailed, unpaired *t*-test with or without Welch’s correction. ****p < 0.0001, ***p < 0.001, **p < 0.01, *p < 0.05, ns: not significant. Primer sequences are provided in Supplementary Table 1.

### Bioinformatic data analysis

Statistical analysis: Established standard tests (e.g., for differential expression testing, gene set enrichment) were used throughout. Correction for multiple testing was performed where stated. Details of individual statistical tests are provided in the respective method sections.

Proteomics data analysis: Raw MS data from IP experiments was loaded into Proteome Discoverer (PD, version 2.1.0.81, Thermo Scientific). All MS/MS spectra were searched using MSAmanda v2.1.5.9849^80^. Trypsin was specified as a proteolytic enzyme cleaving after lysine and arginine (K and R), allowing for up to 2 missed cleavages. Mass tolerances were set to ±5 ppm at the precursor and ±15 ppm at the fragment mass level. Peptide and protein identification was performed in two steps. An initial search was performed against the SwissProt database using taxonomy mouse (release 2017_09; 16,903 sequences; 9,540,942 residues), with common contaminants appended. Here, beta-methylthiolation of cysteine was searched as fixed modification, whereas oxidation of methionine, deamidation of asparagine and glutamine and glutamine to pyro-glutamate conversion at peptide N-termini were defined as variable modification. Results were filtered for a minimum peptide length of 7 amino acids and 1% FDR at the peptide spectrum match (PSM) and the protein level using Percolator^81^. Additionally, an Amanda score of at least 150 was required as well as a minimum PSM-count per protein in at least one sample of 2. Identified proteins were exported and subjected to a second step search considering additional variable modifications. These were: phosphorylation of serines, threonines, and tyrosines, acetylation of lysines and N-termini, ubiquitination (GG) of lysines, mono-, di- and tri-methylation of lysines as well as mono- and di-methylation of arginines. Identifications were filtered using the filtering criteria described above and subjected to label-free quantification using IMP-apQuant^82^. Statistical significance of differentially expressed proteins was determined using a two-sided limma test^83^ (moderated *t*-test with Benjamini–Hochberg correction for multiple testing). TMT fractions of time-resolved phosphoproteomics samples after Mek inhibition were processed similarly but with the following differences in the analysis workflow. Here, mascot^84^ (version 2.2.7, Matrix Science, UK) was used as a database search engine. Up to 3 missed cleavages were considered. Mass tolerance was set to 30 mmu at the precursor and 10 ppm at the fragment mass level. TMT-10plex at lysine and peptide N-termini as well as carbamidomethylation of cysteines was set as fixed modification. Oxidation of methionine, phosphorylation of serine, threonine and tyrosine, deamidation of asparagine and glutamine as well as acetylation of protein N-termini were set as variable modifications. Identified peptides were filtered for 1% FDR at PSM, peptide and protein level using Percolator^81^ and a minimum Mascot Score of 10 was required. ptmRS^85^ was used for phospho site localisation. Spectra were quantified based on reporter ion intensities using the “Reporter Ions Quantifier” node in PD. Identified spectra were exported to R for further processing. All spectra with 5 or more missing reporter ion intensities or when signal intensity of all reporters was below 2000 were removed. Resulting spectra were grouped to peptides based on sequence and phospho site localisation considering phosphorylation sites with >95% site localisation probability as confidently localised. All peptides with at least two-fold regulation compared to untreated samples were subjected to k-means^86^ clustering.

AlphaFold2 interaction screen: All sequences used in the AlphaFold2 interaction screen were retrieved from UniProt and full-length Erf (UniProt: P70459) was used as bait for all models. See Supplementary Data 2 for details on the iPTM and PTM values of each model. Due to memory limitations of our GPU servers, with sequence pairs >1500 amino acids, the second sequence was split according to domain structures as indicated in the UniProt entry. AlphaFold2-Multimer predictions were performed with ColabFold v.1.3.0 using default parameters and databases, as described in detail by Mirdita et al.^87^ and Evans et al.^17^. ColabFold offers an accelerated prediction of protein structures and complexes by combining the fast homology search of MMseqs2 with AlphaFold2 or RoseTTAFold. ColabFold is an open-source software available at https://github.com/sokrypton/ColabFold, and its environmental databases are available at https://colabfold.mmseqs.com.

RNA quantification (QuantSeq): Libraries generated with the QuantSeq FWD kit (Lexogen) were sequenced on an Illumina HiSeq2500v4 at single read 50 bp. Reads were trimmed with bbduk (version 38.86). Quality control was performed using fastQC (0.11.9)^88^. Transcripts were mapped to the mouse reference genome mm10 using STAR (2.7.3a)^89^. After indexing with samtools (1.10) reads in genes were counted using htseq (version 0.11.2)^90^. Differential expression analysis was conducted using DESeq2 (1.24.0)^91^. DESeq2 was performed using the Wald test and Benjamini–Hochberg correction for multiple testing. MA plots were generated with ggplot2 (3.3.3) and the heatmaps using pheatmap (1.0.12)^92^. Z-scores of variance-stabilised counts were used for the heatmaps. Variance-stabilisation transformed expression data were scaled by row and distances were measured by dist from the stats package (4.1.0). fviz_nbclust of the factoextra (1.0.7) package was used to determine optimal cluster number by the elbow method. Agnes of the cluster (2.1.4) package was used for hierarchical clustering by the “ward” method. dendsort (0.3.4) was used to re-sort heatmaps. Median expression line plots and boxplots were generated with ggplot2 using previously computed scaled vst counts and log2fold changes (both based on DESeq2 output). Significance levels in boxplots were computed using ggplot2 stat_compare_means with Wilcoxon rank sum test.

Chromatin Immunoprecipitation Sequencing: Libraries were sequenced on the Illumina HiSeq2500 v4 at single read 50 bp or on the Illumina NovaSeq 6000 SP1 at paired-end 50 bp aiming for a sequencing depth of 20–40 million reads/sample. Raw reads were trimmed with CUTADAPT (2.8) and aligned to the mouse reference genome mm10 with bowtie 2 (2.3.5.1)^93^. Alignments were further processed using samtools (1.10). Peaks were called using MACS2 (2.2.7.1) with a *p*-value cutoff of 0.1^94^. Separate replicated inputs were used for Ncor1, Ncor2 and H3K27ac, H3K27me3 and H3K4me3 ChIP-seq, respectively. For paired-end sequencing all software was run in paired-end mode. High confidence peaks were generated by IDR filtering (1.2), using a cut-off of FDR < 0.05 across all replicates via the ChIPpeakAnno package (3.26.0)^95^. IDR filtered peaks were used for downstream analysis. Motif enrichment analysis was done with HOMER using findMotifGenome.pl using the cumulative binomial probability method^96^. Overlaps between ChIP-seq peaks were computed by findOverlapsOfPeaks and makeVennDiagram. For visualization we generated RPKM normalised coverage files for Tbl1x, Erf-V5 with deeptools (3.5.0) and bamCoverage^97^, while ChIP-seq data for histone modifications, Ncor1, and Ncor2 were additionally normalised by Drosophila spike-in reads. Scaling factors for the spike-in were calculated as in^98^. ChIP-seq tracks were visualised using Galaxy and UCSC genome browser custom tracks^99,100^. Published ChIP-seq data sets of Lee et al., Latos et al., and Adachi et al. were mapped to mm10 as described above^31,43,76^. For the data of Lee et al. the replicates were merged after mapping as in the original study. After peak calling with MACS2 default parameters, a consensus peak-set including all detected peaks was generated with DiffBind. Bigwig scores in the consensus peak-set were counted with multiBigwigSummary and Spearman correlation was computed with plotCorrelation of deeptools. Datasets passing a correlation cutoff of 0.4 with any ChIP of Ncor1, Ncor2 or Tbl1x were regarded as potential coregulators of TNN. The correlation matrix derived by deeptools was visualised with pheatmap (1.0.12).

Differential ChIP enrichment analysis: Differential ChIP enrichment analysis was done using DiffBind (3.2.3)^101^. Peaks passing the IDR cut-off of 0.01 were used to generate a merged peak-set including the peaks of all factors in all tested conditions (SR and PD) by the dba.peakset consensus retrieval function as a reference peak-set for the differential binding analysis of Ncor1, Ncor2, and Tbl1x. Pre-calculated spike-in scaling factors were used for normalization (see above). DiffBind was run in DESeq2 mode with a Wald test and Benjamini–Hochberg multiple testing correction. To reduce the impact of conservative significance cut-offs, which underestimated the near global changes of NCoR1/2 binding after Erf depletion, we removed the significance cut-off (FDR < 0.05) for the initial output of the LFC matrix of all factors. Then, we first filtered by LFC for specific behaviour in Erf-KO vs WT during differentiation (e.g. TNN loss = Ncor1 & Ncor2 & Tbl1x LFC < 0) and second filtered by FDR < 0.5 for at least one factor. The remaining regions were included in the boxplot of Fig. 3h and in the heatmap of Fig. 3i. For histone modifications IDR filtered peaks were used as reference. Correlation heatmaps of reads in ChIP-seq peaks were generated using DiffBind considering all peaks found in at least one IDR filtered peak-set. MA plots showing differential enrichment of ChIP-seq signals were constructed using the internal DiffBind function.

Integration of ChIP-seq and QuantSeq data: Feature distribution was determined, and peaks were annotated with ChIPseeker (1.26.0) with the following priorities: Promoter, 5'UTR, 3'UTR, Exon, Intron, Downstream, Intergenic^102^. Gene symbols were retrieved using biomaRt^103^ and gene overlaps between ChIP-seq and/or QuantSeq data were plotted using Eulerr (6.1.0) or UpSetR (1.4.0)^104,105^. After extraction of specific gene sets functional enrichment analysis was conducted as described below. For Spearman correlation analysis (Fig. 5), LFC of the ChIP-seq signal over peaks was computed with DiffBind (3.2.3)^101^ and matched with up and downregulated genes, respectively.

Functional Enrichment Analysis: Over-representation analysis (ORA) for GO terms and KEGG related terms was conducted with clusterProfiler^106^ and plotted with the internal plotting function or ggplot (3.3.3). ClusterProfiler uses a one-sided Fisher’s exact test for ORA. *P*-values of enrichment analyses were adjusted for multiple testing by the Benjamini–Hochberg method and only terms with an adjusted *P*-value <0.05 were considered. Gprofiler2 was used to test for enrichments across GO, KEGG, ReactomePA, and WikiPathway databases by a one-sided Fisher’s exact test corrected for multiple testing by the tailored g:SCS algorithm^107,108^. Data was plotted with the built-in plotting function “gostplot”.

(Super) enhancer analysis: H3K27ac replicates and inputs were merged and peaks in self-renewal and differentiation were called using MACS2 with default parameters. Enhancers in self-renewal and differentiation were identified using ROSE^34,109^ with a stitching distance of 12.5 kb and TSS exclusion size of 2.5 kb and ranked based on their H3K27ac signal. For each enhancer the closest gene, as determined by ROSE_geneMapper.py, was used for downstream analyses. Enhancers were split into super enhancers (SEs) and regular enhancers (REs) based on ROSE classification. For the ETNN/enhancer overlaps, all enhancers were overlapped with ETNN peaks and split into SEs and REs based on ROSE classification. The resulting gene sets were overlapped with expression data from Fig. 4, the regulated enhancers (in differentiated Erf-KO compared to WT cells) of each group were considered as Erf-dependent enhancers.

Bulk QuantSeq decomposition: Bulk QuantSeq data (this study) and downloaded snRNAseq data from Marsh and Blelloch^25^ were converted into ExpressionSet objects and decomposed by BisqueRNA with the default parameters of Reference based decomposition. Resulting fractions of identity were averaged between replicates and plotted with ggplot2.

### Reporting summary

Further information on research design is available in the Nature Portfolio Reporting Summary linked to this article.

## Supplementary information

Supplementary Information

Description of Additional Supplementary Files

Supplementary Dataset 1

Supplementary Dataset 2

Supplementary Dataset 3

Supplementary Dataset 4

Supplementary Dataset 5

Supplementary Dataset 6

Reporting Summary

## Data Availability

Accession codes. The raw and processed next-generation sequencing datasets were deposited at the NCBI Gene Expression Omnibus (GEO) repository under accession number: GSE199024 [https://www.ncbi.nlm.nih.gov/geo/query/acc.cgi?acc=GSE199024]. The mass spectrometry proteomics data have been deposited to the ProteomeXchange Consortium via PRIDE^110^ partner repository with the dataset identifier PXD037892. Source data are provided with this paper.

## Source data

Source Data
