# Supplementary material for: The Fgf/Erf/NCoR1/2 repressive axis controls trophoblast cell fate
Source: Nat Commun. 2023 May 4;14:2559. doi: 10.1038/s41467-023-38101-8 (PMC10193302; doi:10.1038/s41467-023-38101-8)
Supplement: Supplementary file 1 — Supplementary Information [file 41467_2023_38101_MOESM1_ESM.pdf]

Supplementary Information

Lackner et al.,

The Fgf/Erf/NCoR1/2 repressive axis controls trophoblast cell fate

List of Material provided:

- Supplementary Figure 1
- Supplementary Figure 2
- Supplementary Figure 3
- Supplementary Figure 4
- Supplementary Figure 5
- Supplementary Figure 6
- Supplementary Figure 7
- Supplementary Figure 8 full-size WB scans
- Supplementary Table 1

Supplementary Figure 1

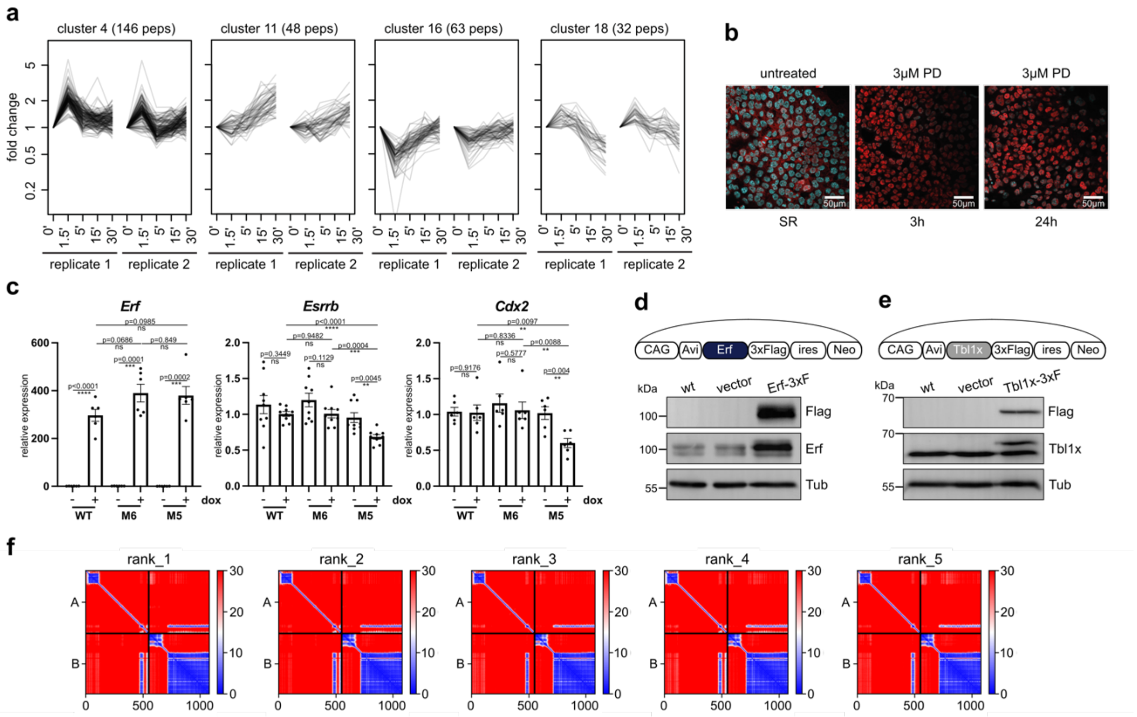

Supplementary Figure 1. Erf interacts with the NCoR1/2 complex in differentiating TSCs.

(**a**) Selected clusters resulting from the phosphoproteomic analysis of TSCs treated for 1.5, 5, 15, and 30 minutes with 3μM Mek inhibitor PD0325901 (PD), compared to the untreated (0’) and displayed as fold change. Analysis based on two biological replicates (n=2). (**b**) Immunofluorescence (n=1) showing localisation of Erf (red) in self-renewal (SR) and after 3h and 24h of PD treatment. DAPI (blue) marks the nuclei. (**c**) RT-QPCR analysis of Erf-KO cell lines carrying doxycycline (dox) inducible transgenes of Erf WT, M5 (S21A, S185A, S190A, S534A, S327A), and M6 (S161A, T529A, S246A, S251A, T357A, T148A) phospho-mutants cultured in self-renewal conditions for 24h in the presence (+dox) or absence (-dox) of dox. The bars represent a mean of three biological replicates (n=3) with S.E.M., expression in WT Erf +dox was set to 1. Statistical significance was determined using unpaired two-tailed t test with Welch’s correction: ****p<0.0001, ***p<0.001, **p<0.01, *p<0.05, ns: not significant. (**d,e**) Schematic depiction of the Erf-3xFlag and Tbl1x-3xFlag expression vectors and Western blot analysis of WT, Erf-3xFlag, Tbl1x-3xFlag and vector control lines probed with indicated antibodies. Representative of three (n=3) biological replicates. (**f**) Predicted aligned error (PAE) plots for the five AlphaFold2 prediction models for the Erf–Tbl1x interaction. The PAE corresponds to the expected error in the position of one residue with respect to another residue in a model (in Angstroms). A short C-terminal region of Erf at around position 480-490 shows

low PAE values (high confidence) to the WD40 domain of Tbl1x (position 780-1070 in the combined sequence), indicated by the blue bar.

Supplementary Figure 2

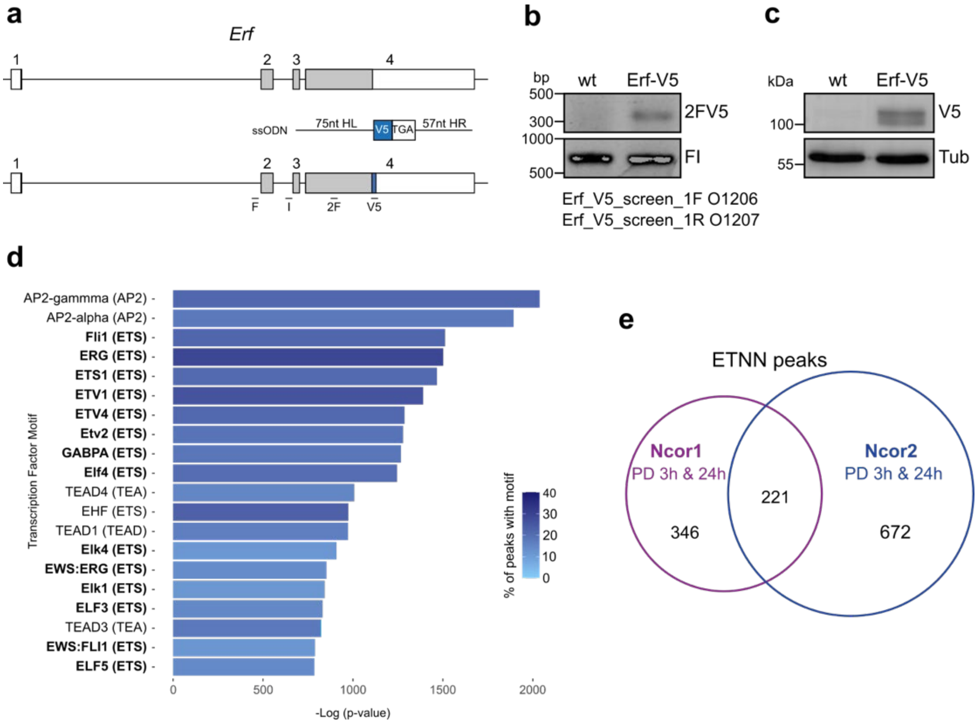

Supplementary Figure 2. Erf co-occupies NCoR1/2 target regions.

(**a**) Generation of the *Erf-V5* knock-in allele. *Erf* locus with exons (rectangles), coding sequence (grey) and genotyping primers is depicted. Single-stranded oligodeoxynucleotide (ssODN) donor template contained 75 nucleotide-(nt)-long left homology (HL), 57nt right homology (HR) and the V5 sequence followed by a stop (TGA) codon. (**b**) PCR genotyping of WT and Erf-V5 lines using indicated primer pairs (see (a)). Representative of two (n=2) biological replicates. (**c**) Western blot analysis of WT and Erf-V5 protein lysates probed with anti-V5 and anti-Tubulin (Tub) antibodies. Representative of three (n=3) biological replicates. (**d**) Known motifs enriched in the Erf ChIP-seq peaks identified by HOMER. Note that Erf belongs to the ETS family of factors. (**e**) Overlap of PD-specific (called in PD_3h and PD_24h but not in SR) Ncor1 and Ncor2 ETNN target regions.

Supplementary Figure 3

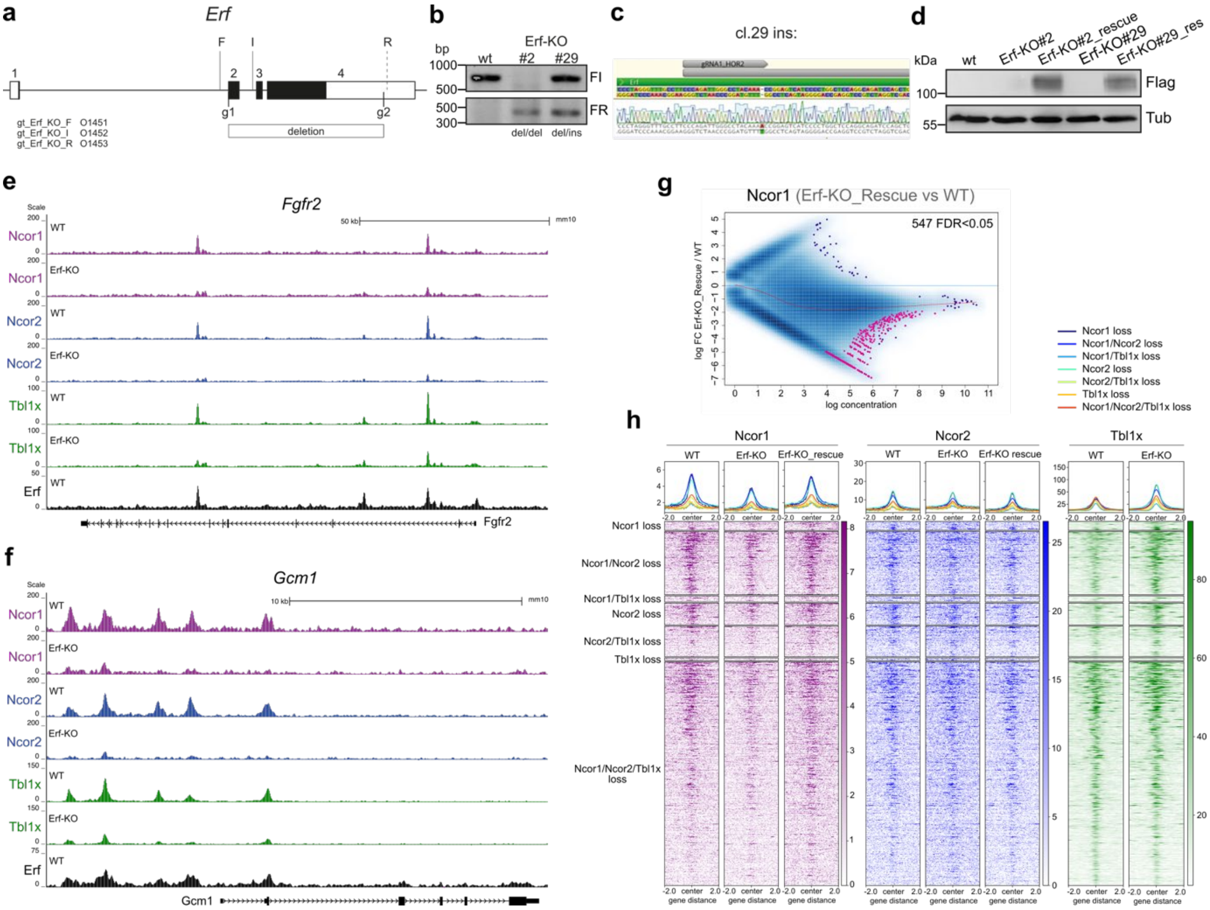

Supplementary Figure 3. Erf recruits the NCoR1/2 complex to its target regions.

(**a**) Generation of the Erf-KO line. Schematic representation of the *Erf* locus with exons (rectangles), coding sequence (black), genotyping primers and guide (g)RNAs. (**b**) PCR genotyping of Erf-KO clone 2 (#2, homozygous deletion between g1-g2) and clone 29 (#29, deletion and insertion) along WT control with indicated primer pairs. Representative of two (n=2) biological replicates. (**c**) Sanger sequencing verification of an insertion in Erf-KO#29. (**d**) Western blot analysis of protein lysates from WT, Erf-KO#2, Erf-KO#2_rescue (Erf-KO#2 rescued with Erf-3xFlag construct from S1d), Erf-KO#29, Erf-KO#29_rescue probed with indicated antibodies. Representative of three (n=3) biological replicates. (**e, f**) Genome browser tracks of Erf, Tbl1x, Ncor1, and Ncor2 signal at the *Fgfr2* (e) and *Gcm1* (f) locus in WT and Erf-KO TSCs after 24h of PD treatment. (**g**) MA plot of Ncor1 binding sites differentially enriched in Erf-KO_rescue vs WT TSCs after 24h PD treatment. Log2 fold change is plotted as a function of the log normalised ChIP-seq reads. (**h**) Heat maps of Ncor1, Ncor2 and Tbl1x ChIP-seq signal in regions with specific or combinatorial reduction of Ncor1,

Ncor2, and Tbl1x binding in differentiating Erf-KO (as shown in main Figure 3a-c), displayed in self-renewing WT, Erf-KO, and Erf-KO_rescue lines.

Supplementary Figure 4

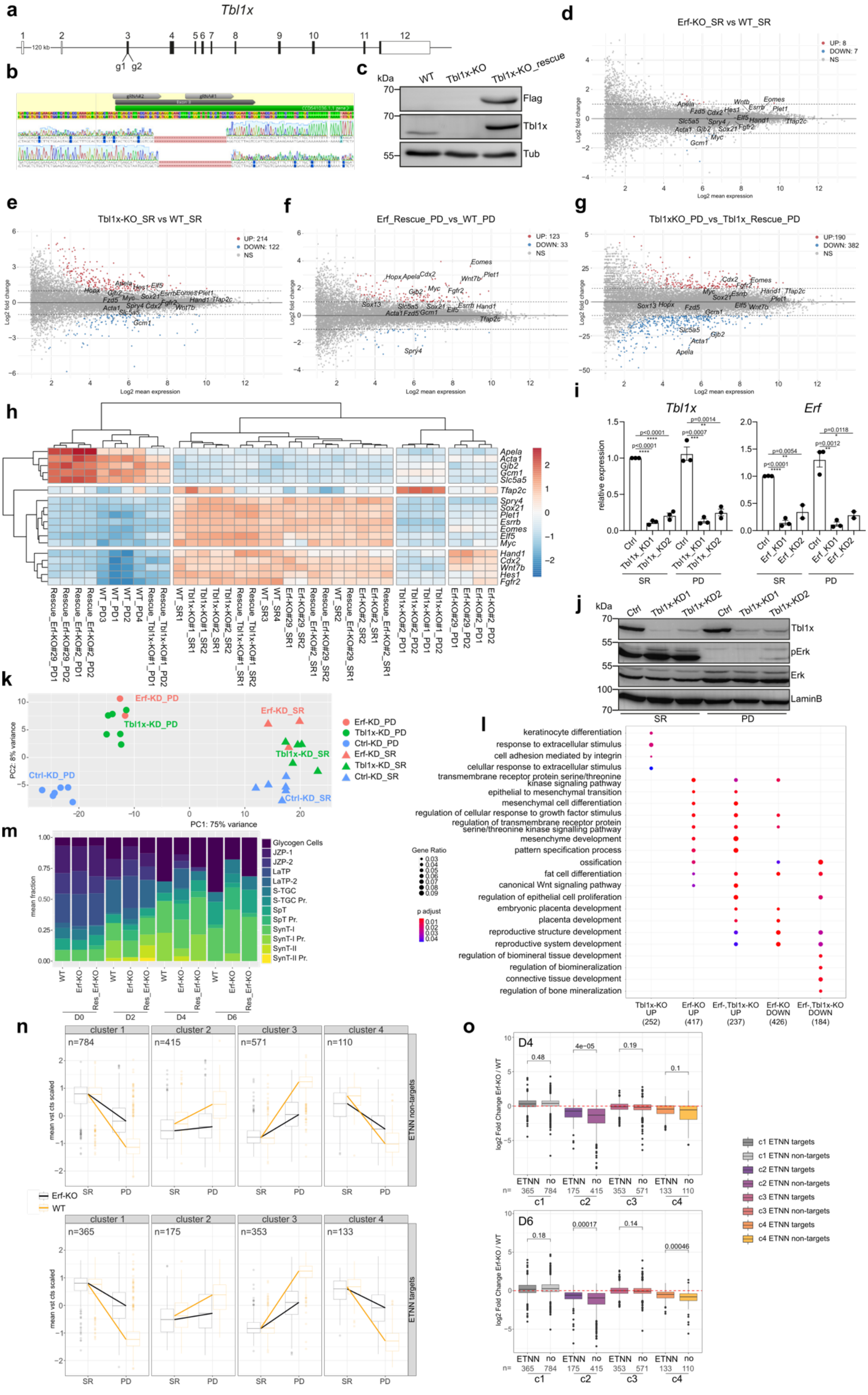

Supplementary Figure 4. Erf/NCoR1/2 controls expression of key trophoblast genes. (**a**) Generation of the Tbl1xKO line. *Tbl1x* locus with exons (rectangles), coding sequence (black), and guide (g) RNAs indicated. (**b**) Sanger sequencing verification of Tbl1x-KO. (**c**) Western blot analysis of protein lysates from WT, Tbl1x-KO and Tbl1x-KO_rescue (Tbl1x-KO rescued with the Tbl1x-3xFlag construct from S1e) lines probed with indicated antibodies. Representative of three (n=3) biological replicates. (**d**) MA plot of differentially expressed genes between Erf-KO (n=4) and WT (n=4) lines in self-renewal (SR) conditions. Analysis revealed 8 up-regulated and 7 down-regulated genes in Erf-KO lines compared to WT. (**e**) MA plot of differentially expressed genes between Tbl1x-KO (n=4) and WT (n=4) lines in self-renewal (SR) conditions. (**f**) MA plot of differentially expressed genes between Erf-KO_rescue (n=4) and WT (n=4) lines in differentiation (PD) conditions. (**g**) MA plot of differentially expressed genes between Tbl1x-KO_rescue (n=2) and WT (n=4) lines in differentiation (PD) conditions. (d-g) Analysis cut-off: |log2FC|>1, p adj <0.05, Wald-test with Benjamini-Hochberg correction. Significantly up- (UP, red) and down-regulated (DOWN, blue) genes are indicated in red and blue, respectively. NS: not significant. (**h**) Clustered heatmap based on the QuantSeq analysis, depicting row z-score of the variance-stabilised transformed expression values of selected genes in WT, Erf-KO, Erf-KO_rescue, Tbl1x-KO, and Tbl1x-KO_rescue lines in self-renewal (SR) and after 24h PD0325901 (PD) treatment. Numbers indicate biological and technical replicates, respectively. (**i**) RT-QPCR expression analysis for Erf and Tbl1x of control (Ctrl), Erf-depleted (Erf-KD1 and Erf-KD2), and Tbl1x-depleted (Tbl1x-KD1 and Tbl1x-KD2) lines in self-renewal (SR) and after 24h of PD treatment. The bars represent a mean of either two (n=2, Erf-KD2) or three (n=3) biological replicates with S.E.M. Statistical significance was determined using unpaired two-tailed t test: ****p<0.0001, ***p<0.001, **p<0.01, *p<0.05. (**j**) Western blot analysis of protein lysates of control (Ctrl) and Tbl1x-depleted (Tbl1x-KD1 and Tbl1x-KD2) lines in self-renewal (SR) and after 24h of PD treatment probed with indicated antibodies. Representative of three (n=3; anti-Tbl1x, anti-LaminB) and one (n=1; anti-pErk, anti-Erk) biological replicates. (**k**) Principal component analysis plot based on global gene expression (QuantSeq) in WT, Erf-KD, and Tbl1x-KD lines in self-renewal (SR, triangle) and after 24h of PD treatment (circle). (**l**) Gene ontology overrepresentation analysis of the uniquely and commonly deregulated genes in 24h differentiated (PD) Erf-KO and Tbl1x-KO cells compared to WT. (**m**) Fractions of cell identities of deconvoluted bulk RNA-seq data of WT and Erf-KO cells along a 6-day time course after withdrawal of Fgf/CM (WD). Fractions were determined with BisqueRNA by reference-based decomposition using data from Marsh and Belloch[1]**.** Each bulk RNA-seq sample gets a fraction of similarity with all placental cell types earlier defined by the clustering in Marsh and Belloch[1]. (**n**) Aggregated gene expression (median) of the 4 top variance clusters in WT and Erf-KO

cells along the 6-day WD differentiation time course. Box boundaries show the 25th to 75th percentile with the median as centre and whiskers representing the calculated maximum and minimum. Outliers are depicted by the dots. (**o**) Box plots showing the log2 fold changes of clusters separated by ETNN and non-ETNN targets in differentiating Erf-KO compared to WT cells at d4 and d6 of WD. P-values of the Wilcoxon test are indicated. Box boundaries show the 25th to 75th percentile with the median as centre and whiskers representing the calculated maximum and minimum. Outliers are depicted by the dots.

Supplementary Figure 5

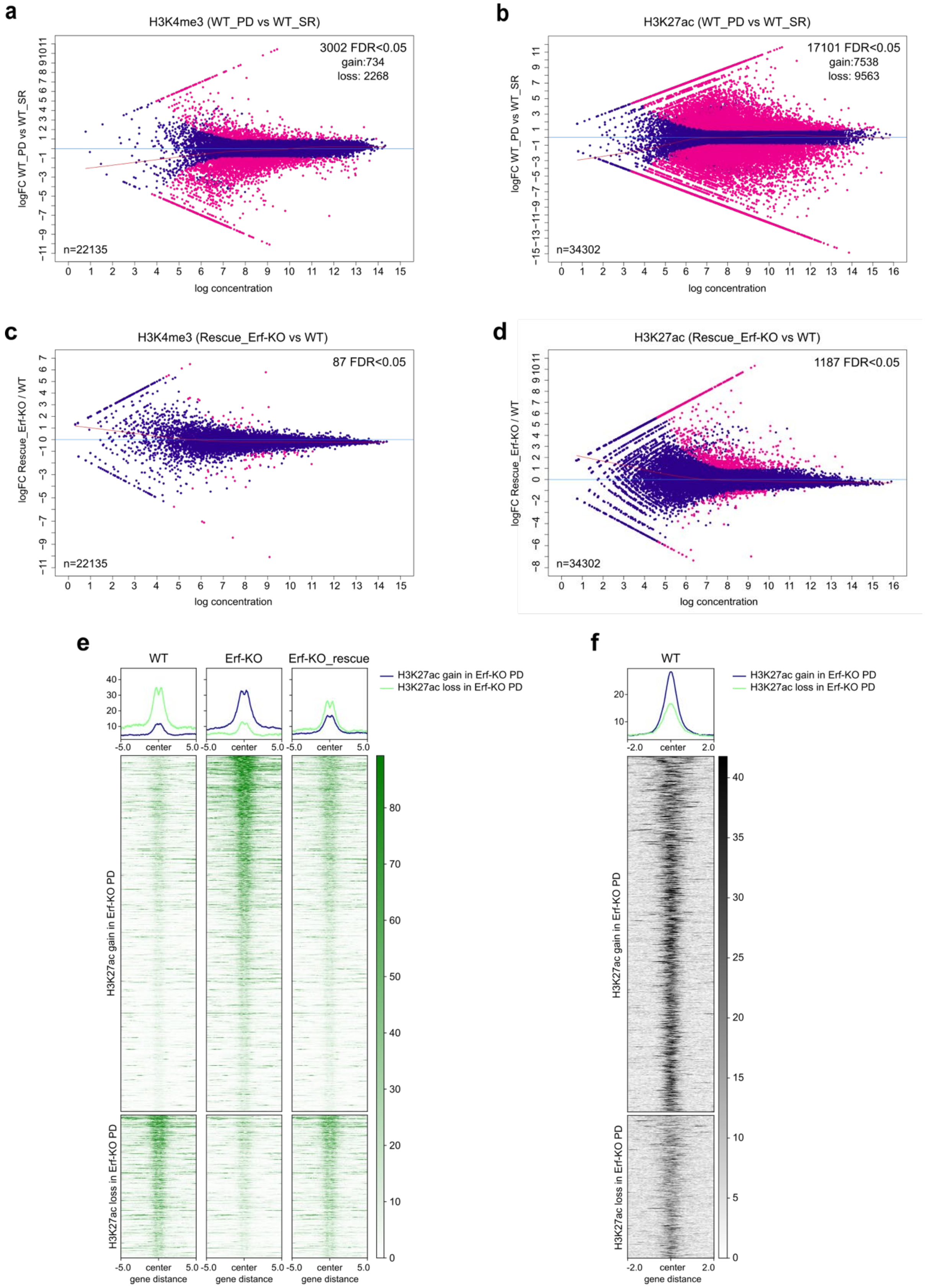

Supplementary Figure 5. Erf disruption affects H3K27ac on target genes upon TSC differentiation.

(**a**) Differentially enriched H3K4me3 regions in differentiated (PD) TSCs (n=2) compared to self-renewing (SR) WT TSCs (n=2). (**b**) Differentially enriched H3K27ac regions in differentiated (PD) TSCs (n=2) compared to self-renewing (SR) WT TSCs (n=2). (**c**) Differentially enriched H3K4me3 regions in differentiated (PD) Erf-KO_rescue TSCs (n=2) compared to WT TSCs. (**d**) Differentially enriched H3K27ac regions in differentiated Erf-KO_rescue TSCs (n=2) compared to WT TSCs (n=2). (a-d) Cut-off: $|log2FC|>1$, p adj <0.05, Wald-test with Benjamini-Hochberg correction. (**e**) Heatmap and average plot of the H3K27ac ChIP-seq signal on the Erf/Tbl1x/Ncor1/Ncor2 (ETNN)-bound regions differentially enriched in H3K27ac in differentiated Erf-KO cells compared to differentiated WT cells. (**f**) Heatmap and average plot of the Erf-V5 ChIP-seq signal on the ETNN regions differentially enriched in H3K27ac in differentiated Erf-KO cells compared to differentiated WT cells (PD).

Supplementary Figure 6

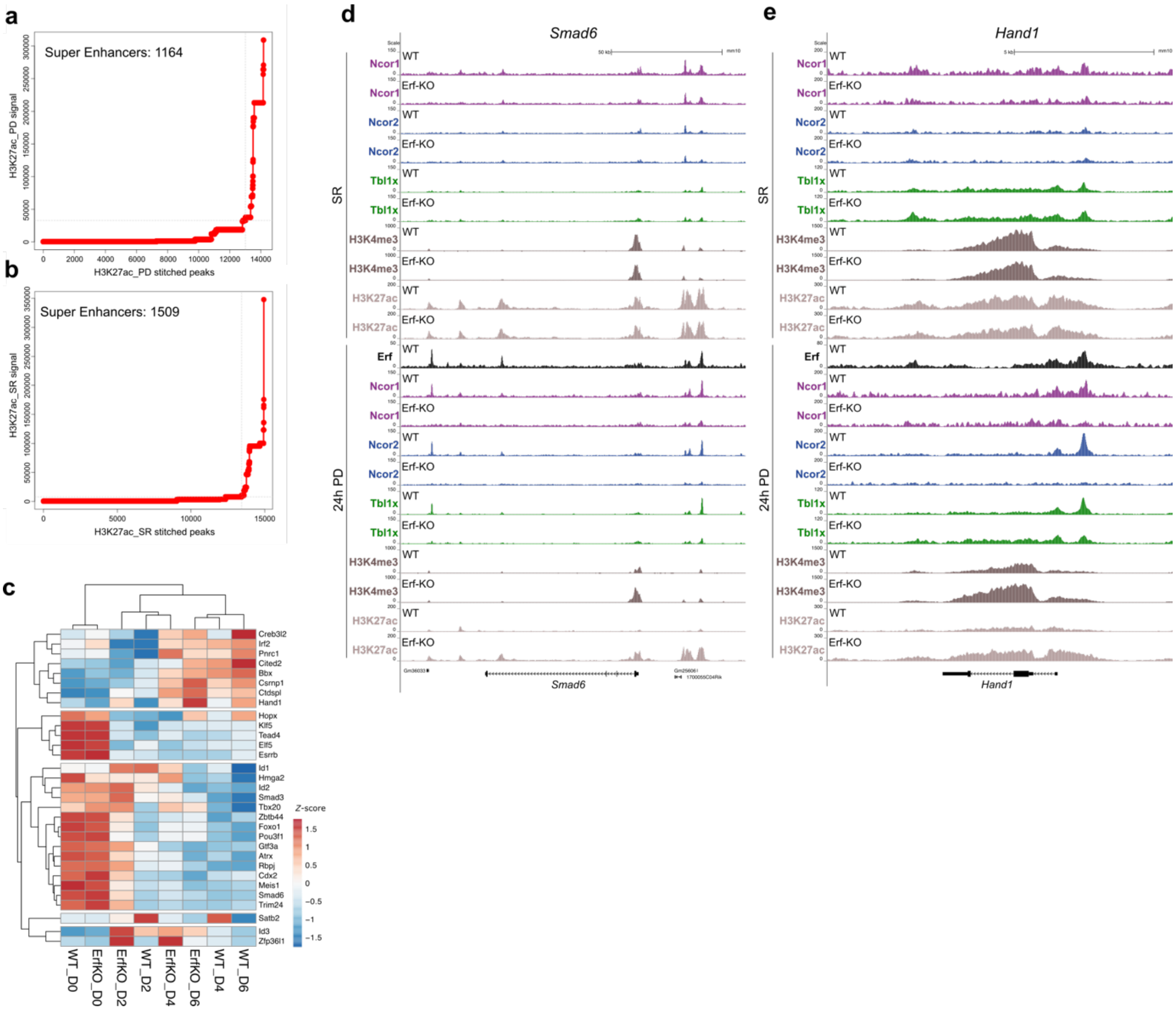

Supplementary Figure 6. Erf/NCoR1/2 regulates key super enhancers during TSC differentiation.

(**a,b**) Line plot showing the H3K27ac signal in (a) differentiating and (b) self-renewing WT TSCs as a function of length of stitched H3K27ac peaks. (**c**) Heatmap of Z-scores based on mean variance stabilised counts (n=3) of the 31 ETNN/SE-associated TFs shared between this study and Lee et al.[2] during differentiation induced by withdrawal of Fgf/CM. (**d,e**) Genome browser tracks of Erf, Ncor1, Ncor2, Tbl1x, H3K4me3 and H3K27ac signal at the SE-associated loci (d) *Smad6* and (e) *Hand1* in self-renewing (SR) and differentiating (PD) TSCs.

Supplementary Figure 7

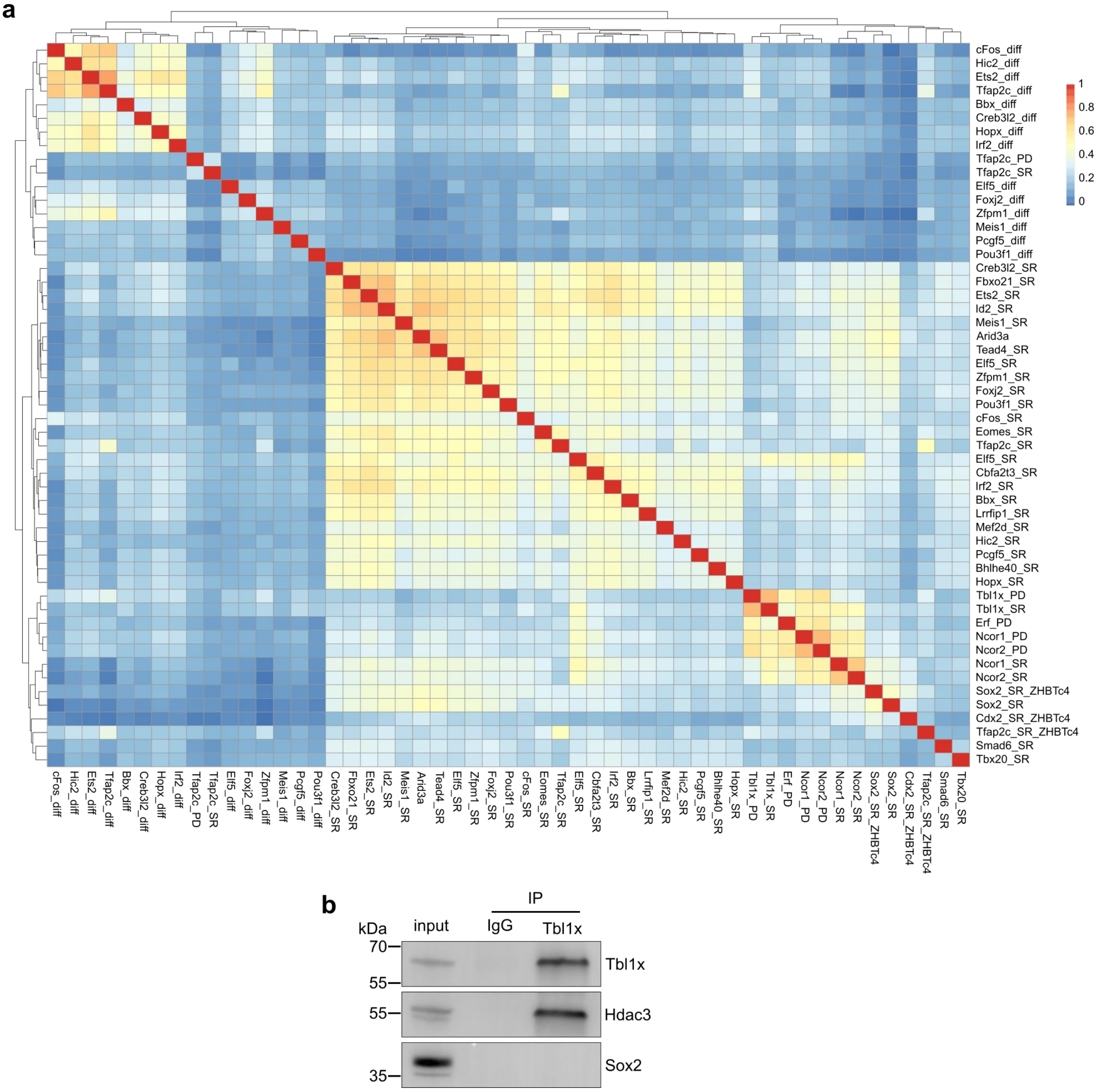

Supplementary Figure 7

(**a**) Heatmap showing a Spearman correlation matrix of the normalised signal of published ChIP-seq data sets (Adachi et al.[3], Latos et al.[4], Lee et al.[2]) and all data sets of this study within the peaks that were identified in all data sets. (**b**) Endogenous Tbl1x immunoprecipitates from TSCs cultured in SR, analysed by Western blot with indicated antibodies. IgG serves as a negative control. Representative of two (n=2) biological replicates.

Supplementary Figure 8

a

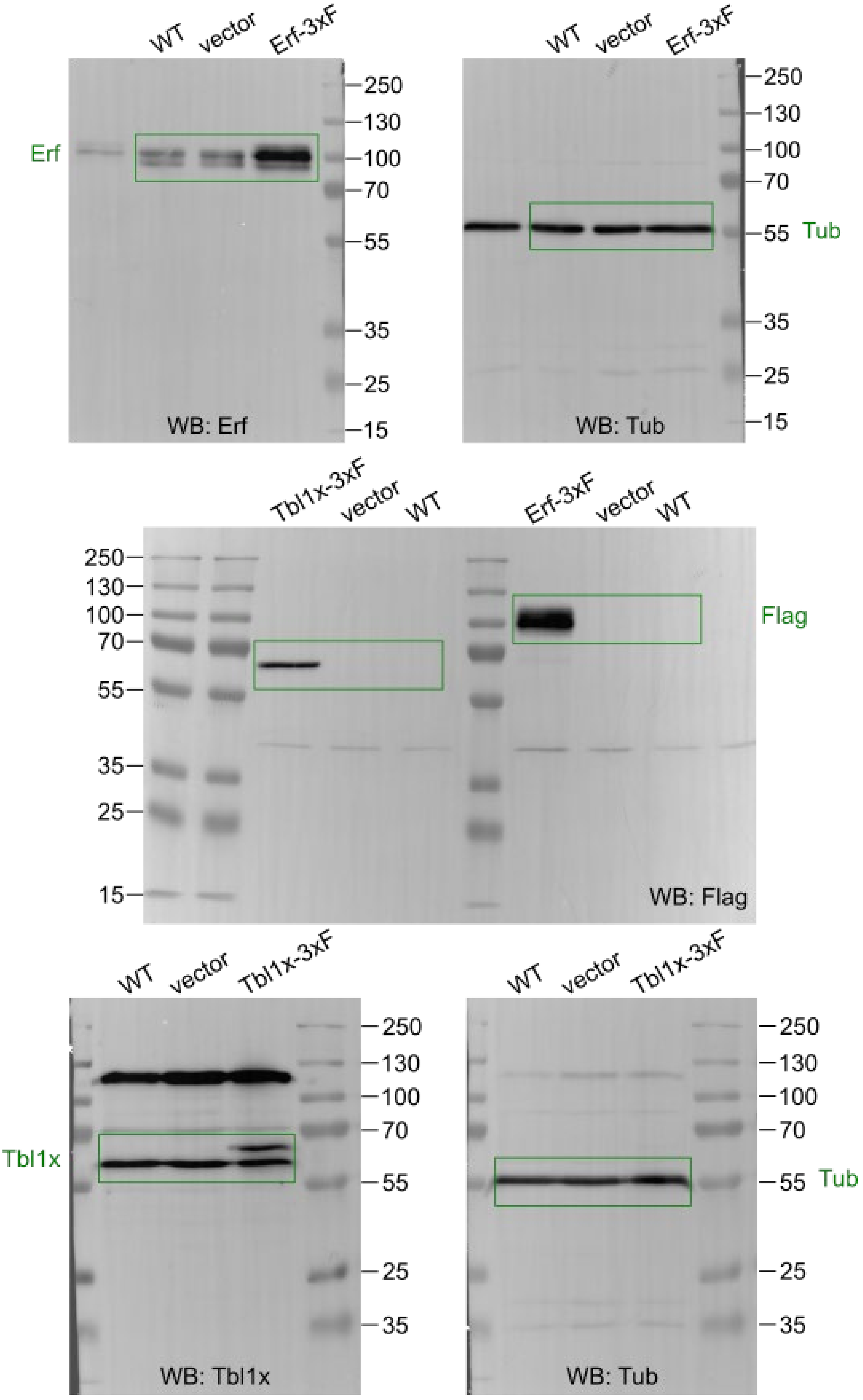

b

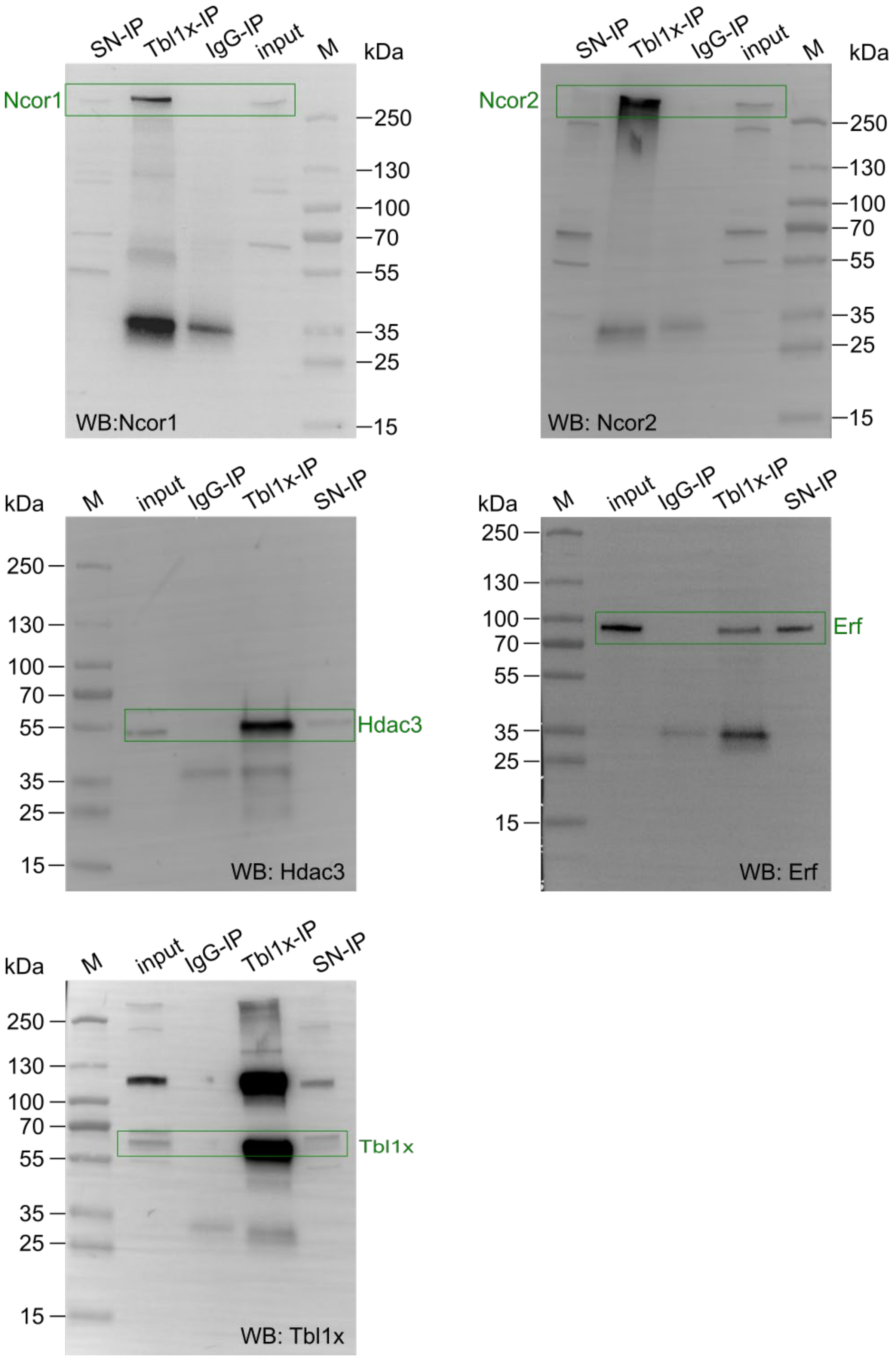
SN-IP
Tbl1x-IP
IgG-IP
input
M
kDa
Ncor1
250
130
100
70
55
35
25
15
WB:Ncor1
Ncor2
WB: Ncor2
Hdac3
WB: Hdac3
Erf
WB: Erf
Tbl1x
WB: Tbl1x

c

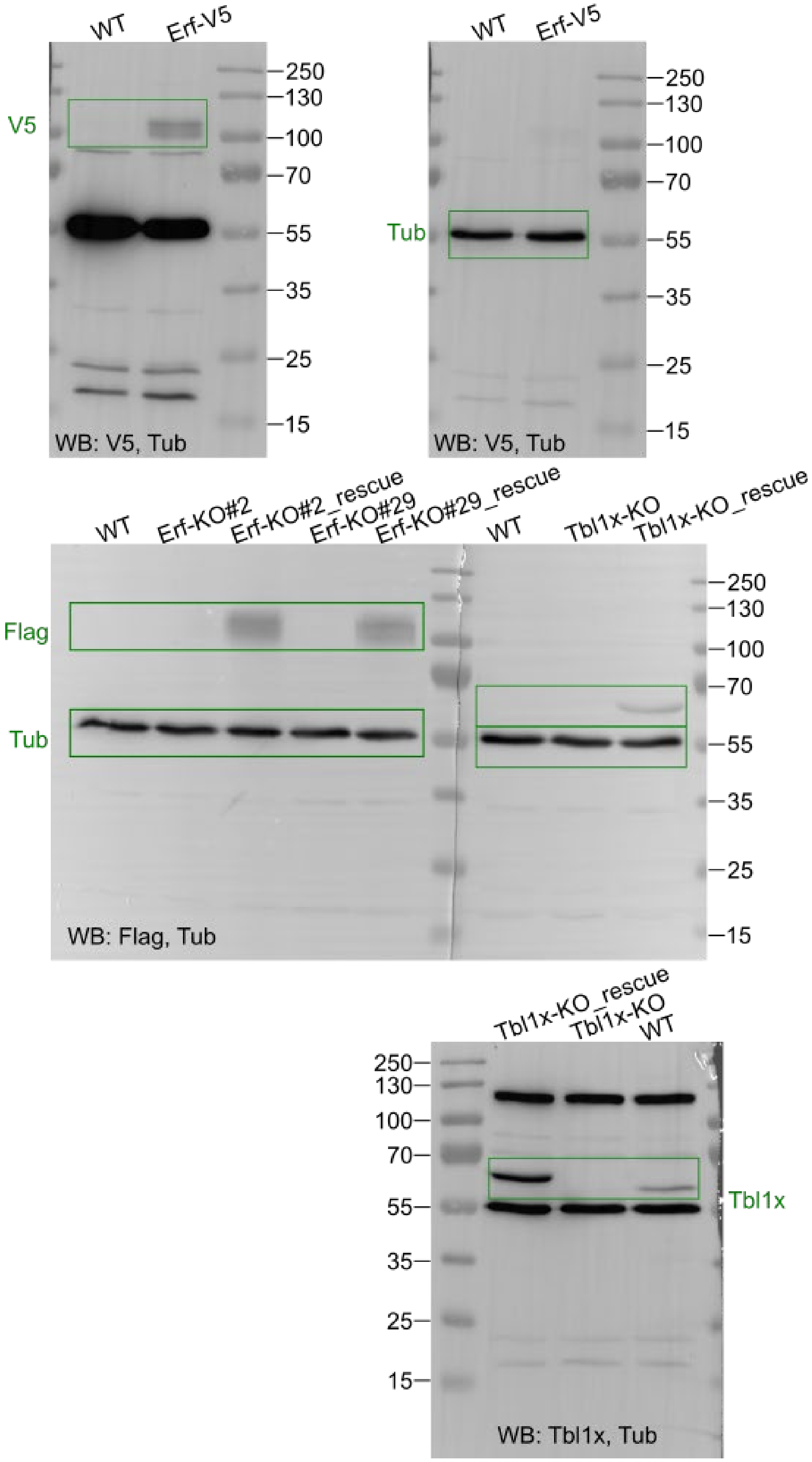
WT
Erf-V5
V5
250
130
100
70
55
35
25
15
WB: V5, Tub
WT
Erf-V5
Tub
250
130
100
70
55
35
25
15
WB: V5, Tub
WT
Erf-KO#2
Erf-KO#2_rescue
Erf-KO#29
Erf-KO#29_rescue
WT
Tbl1x-KO
Tbl1x-KO_rescue
Flag
Tub
250
130
100
70
55
35
25
15
WB: Flag, Tub
Tbl1x-KO_rescue
Tbl1x-KO
WT
250
130
100
70
55
35
25
15
Tbl1x
WB: Tbl1x, Tub

d

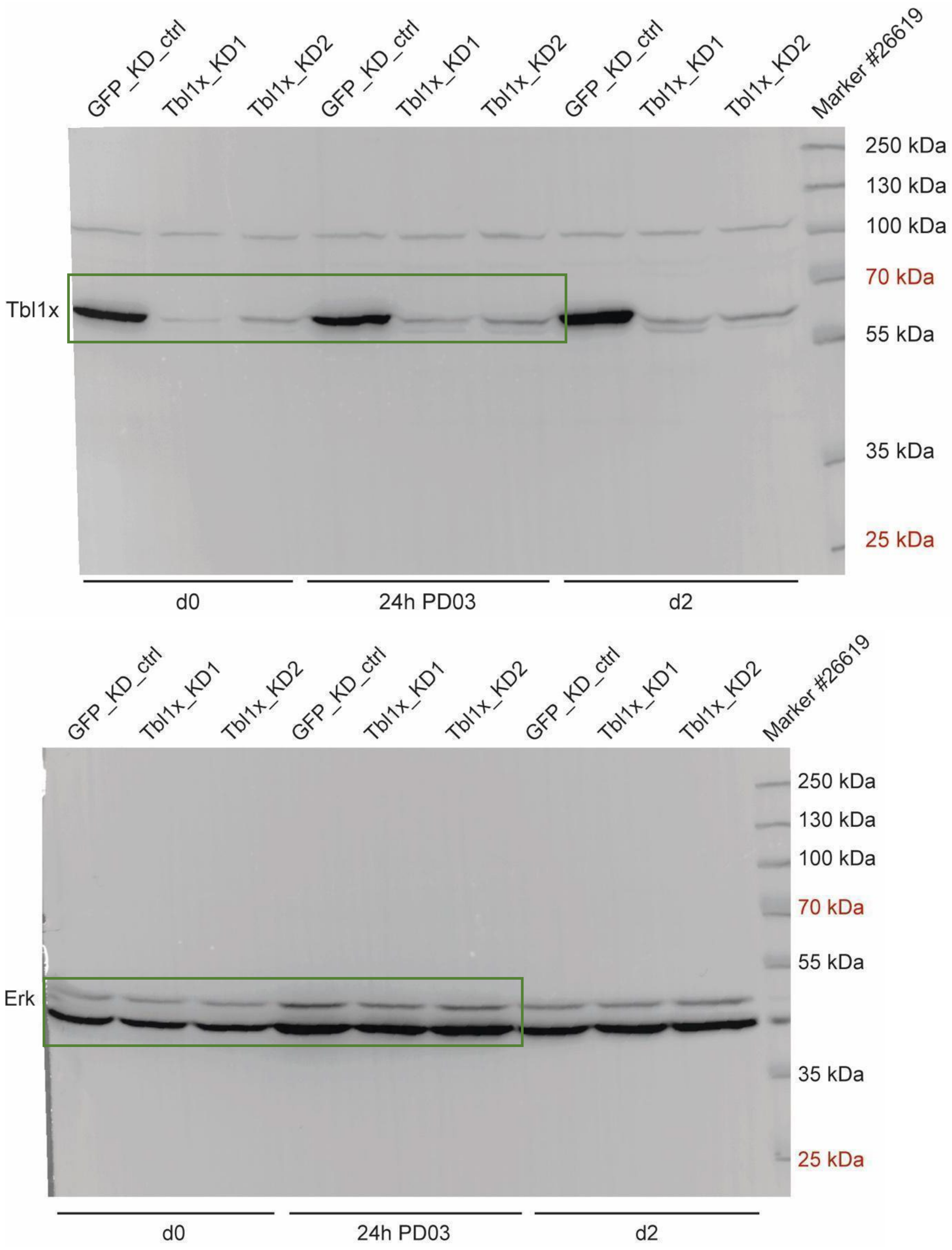
GFP_KD_ctrl
Tbl1x_KD1
Tbl1x_KD2
GFP_KD_ctrl
Tbl1x_KD1
Tbl1x_KD2
GFP_KD_ctrl
Tbl1x_KD1
Tbl1x_KD2
Marker #26619
Tbl1x
250 kDa
130 kDa
100 kDa
70 kDa
55 kDa
35 kDa
25 kDa
d0
24h PD03
d2
GFP_KD_ctrl
Tbl1x_KD1
Tbl1x_KD2
GFP_KD_ctrl
Tbl1x_KD1
Tbl1x_KD2
GFP_KD_ctrl
Tbl1x_KD1
Tbl1x_KD2
Marker #26619
Erk
250 kDa
130 kDa
100 kDa
70 kDa
55 kDa
35 kDa
25 kDa
d0
24h PD03
d2

e

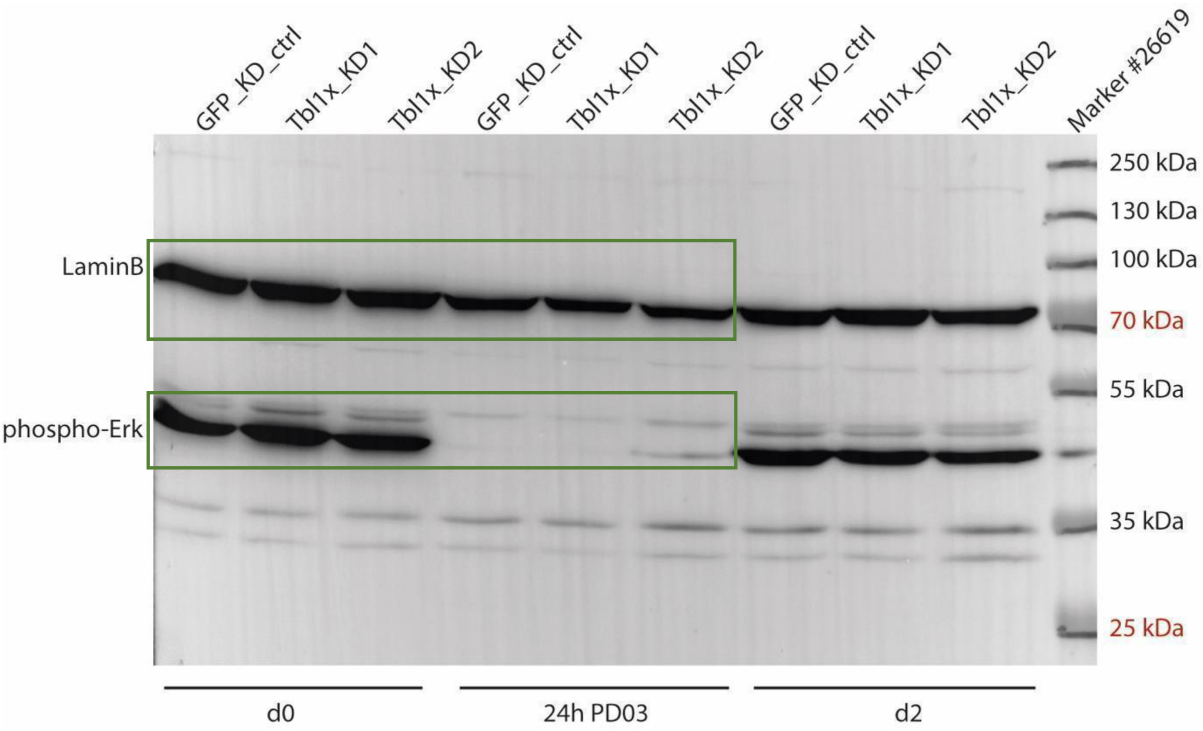
GFP_KD_ctrl
Tbl1x_KD1
Tbl1x_KD2
GFP_KD_ctrl
Tbl1x_KD1
Tbl1x_KD2
GFP_KD_ctrl
Tbl1x_KD1
Tbl1x_KD2
Marker #26619
LaminB
phospho-Erk
250 kDa
130 kDa
100 kDa
70 kDa
55 kDa
35 kDa
25 kDa
d0
24h PD03
d2

f

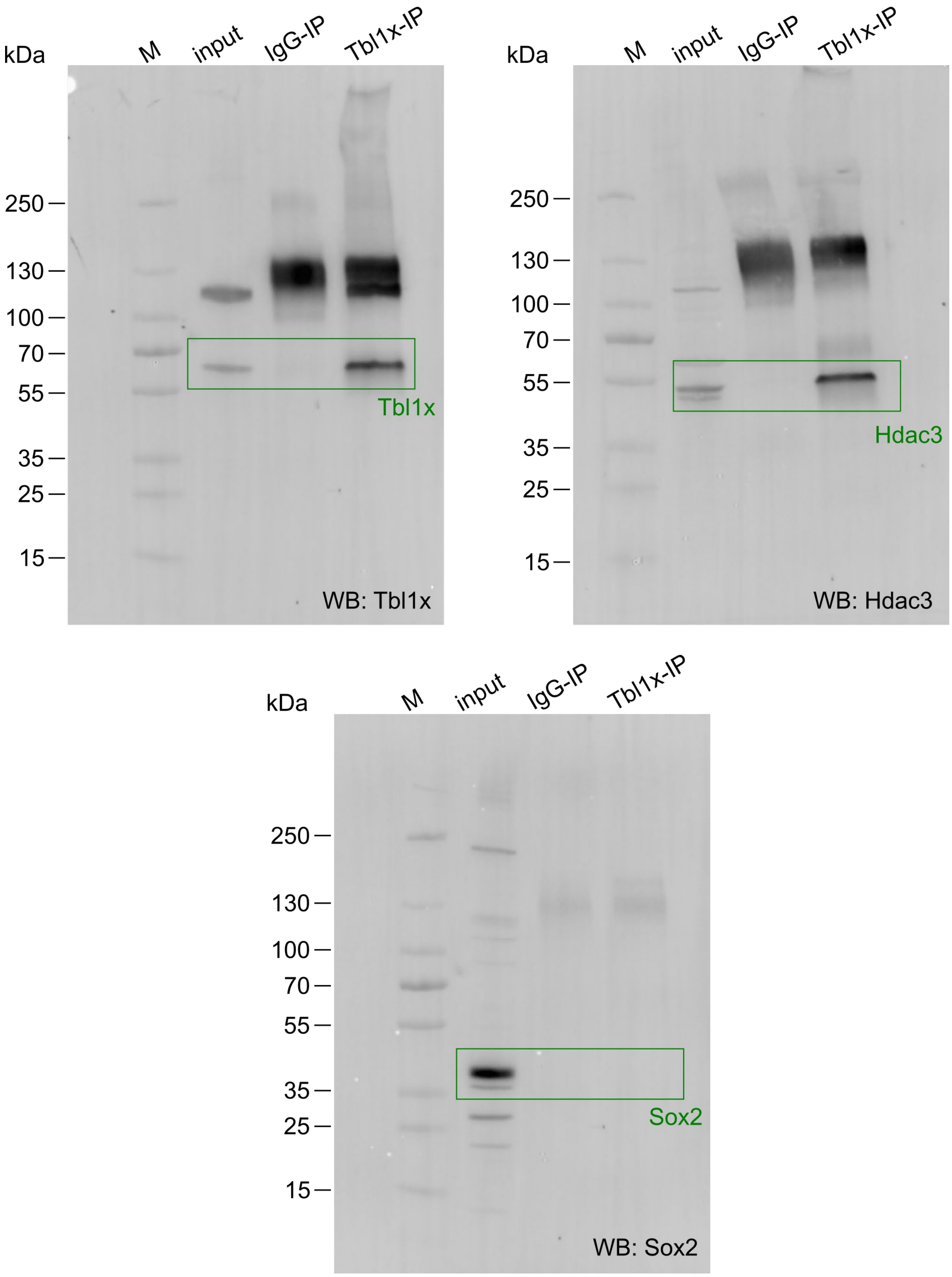
kDa
M
input
IgG-IP
Tbl1x-IP
250
130
100
70
55
35
25
15
Tbl1x
WB: Tbl1x
kDa
M
input
IgG-IP
Tbl1x-IP
250
130
100
70
55
35
25
15
Hdac3
WB: Hdac3
kDa
M
input
IgG-IP
Tbl1x-IP
250
130
100
70
55
35
25
15
Sox2
WB: Sox2

Supplementary Figure 8 Primary scans of Western blots.

(**a**) Primary scans of Western blots as in Supplementary Fig.1d,e. (**b**) Primary scans of Western blots as in Fig.1i. (**c**) Primary scans of Western blots as in Supplementary Fig.2c, 3d,4c. (**d**) Primary scans of Western blots as in Supplementary Fig.4j. (**e**) Primary scans of Western blots as in Supplementary Fig.4j. (**f**) Primary scans of Western blots as in Supplementary Fig.7bj.

Supplementary Table 1

**gRNAs**		
Erf_g1	CCCAGATTGGGCCTACAAAC	
Erf_g2	GCCTAGTTGACCTGTTCAGG	
Tbl1x_g1	TCTGGTATATCGCTACCTCC	
Tbl1x_g2	GATGAGCATTACCAGCGACG	
**shRNAs**		
TRCN0000109356	GCGAGGATATGGAACCTTAAT	Tbl1x
TRCN0000109359	GACATGATGTTCCCAGTAATA	Tbl1x
TRCN0000084155	GTGACTGACATCAGTGACGAA	Erf
TRCN0000084156	GCTATTATTACAACAAGCGCA	Erf
**Erf-V5**		
ssODN	ACGGGGCGGTTAAGGCAGCAAAAGCTCAGGGAGTGGGGTGGTGGGTGGGTGGGGGTG**GGCCCGTGCCCACAGCTCTCACGTAGAATCGAGACCGAGGAGAGGGTTAGGGATAGGCTTACC**GGAATCTCGGTGCTCCAGGGAGAGTTGGGCTGTGGCGTGCTGGAGGTCAGAGCTCACCCGTCGTGGGGTAAGGGG	
crRNA_Erf	GGCCCGTGCCCACAGCTCTC	
**Genotyping primers**		
gt_Erf_KO_F	CCCTCACGCAGATGCCCATA	
gt_Erf_KO_I	AAAGGGTACCCAACAGCCGAG	
gt_Erf_KO_R	AGAGCTGGAGGGCAAAAGCA	
Erf_V5_screen_1F	GGAAGATGGGGAGGTGTTCAA	
Erf_V5_screen_1R	GGAGAGGGTTAGGGATAGGCTT	
**Phosphomutants**		
S21A_Erf_F	ACCGGAGTCAGCCCCTGGCTCCA	
S21A_Erf_R	TTGTAGGCCCAATCTGGGAAGGCAAAC	
S161A_Erf_F	GGATCCCCGAGCTCCACCGGCTT	
S161A_Erf_R	TCAGTGGGGGACAGCACCTC	
S327A_Erf_F	ACAACTACCACCTCGCTCCCCGCGCCTTCC	

S327A_Erf_R	TTGTAGACGCTTTGGGTG	
T357A_Erf_F	GGCACCGGAGGCCCCGCCGGTCC	
T357A_Erf_R	ATGGGCGGCAGTGGGCACTTGTCAGG	
S534A_Erf_F	ACGACGGGTGGCCTCTGACCTCCAGC	
S534A_Erf_R	GGGGTAAGGGGTCCCCCA	
S246/251A_Erf_F	CCTGTGGCACCTTTGGCTGGGCCTGGC	
S246/251A_Erf_R	GAAGGGAGCCAGGGGTTCAGGACCACC	
S185/190A_Erf_F	GACTGTGCTGATGGCACCTCAGAGCTGG AGGAG	
S185/190A_Erf_R	ACTGACTGCGCCTCGGCCCAGGCGTCG	
mErf_F2_mutF	CGAGTGGCATGAGAACCTGT	
mErf_F3_mutF	TTCATCGATATGGGGCTGGC	
mErf_F3_mutR	GAGTCATGGGCAGAGCTGG	
mErf_F4_mutF	TTCTACCCCCTCAGCTCTCC	
mErf_F4_mutR	CCACCACTGCTCTTGTCAGT	
mErf_F5_mutF	GTGCATGCCCCTTAAACTGC	
mErf_F5_mutR	GCCGCTACTTGTCATCGTCA	
**Expression primers**		
Gcm1_1F	GCTCCACAGAGGAAGGCCGC	
Gcm1_1R	GTTGGTGACCGGGAAGCCGC	
mTbl1x-1F	CACAAGTTGCACGGCTCGC	
mTbl1x-1R	GTGTGAGCCACCCTCGTCAC	
mErf-1F	TGAAGACCCCGGCGGACA	
mErf-1R	CCCCCTGCCAAGCGATGAC	
mPbgd (Hmbs)-R	CTGCAGCCTCCTTCCAGGTG	
mPbgd (Hmbs)-F	CGTGGAAGTCCGAGCCAAGG	
mGapdh_1F	ACAACTCACTCAAGATTGTCAGCA	
mGapdh_1R	ATGGCATGGACTGTGGTCAT	
mCdx2_1F	AGTGAGCTGGCTGCCACACT	
mCdx2_1F	GCTGCTGCTGCTTCTTCTTGA	
mEsrrb_1F	AGTACAAGCGACGGCTGG	
mEsrrb_1R	CCTAGTAGATTCGAGACGATCTTAGTCA	

References

1. Marsh, B. & Blelloch, R. Single nuclei RNA-seq of mouse placental labyrinth development. *Elife* **9**, e60266 (2020).
2. Lee, B.-K. *et al.* Super-enhancer-guided mapping of regulatory networks controlling mouse trophoblast stem cells. *Nat Commun* **10**, 4749 (2019).
3. Adachi, K. *et al.* Context-dependent wiring of Sox2 regulatory networks for self-renewal of embryonic and trophoblast stem cells. *Mol. Cell* **52**, 380–392 (2013).
4. Latos, P. A. *et al.* Fgf and Esrrb integrate epigenetic and transcriptional networks that regulate self-renewal of trophoblast stem cells. *Nat Commun* **6**, 7776 (2015).
