# Supplementary material for: The Fgf/Erf/NCoR1/2 repressive axis controls trophoblast cell fate
Source: Nat Commun. 2023 May 4;14:2559. doi: 10.1038/s41467-023-38101-8 (PMC10193302; doi:10.1038/s41467-023-38101-8)
Supplement: Supplementary file 2 — Description of Additional Supplementary Files [file 41467_2023_38101_MOESM2_ESM.pdf]

Description of Additional Supplementary Files:

Supplementary Data 1: Fold changes of phosphorylated peptides associated with genes.

Supplementary Data 2: Alpha Fold IPTM and PTM values.

Supplementary Data 3: ETNN target status, group (Upset plot Fig4e), LFCs and padj in ErfKO and Tbl1xKO PD relative to WT PD.

Supplementary Data 4: Genes of clusters derived by hierarchical "ward" clustering of the 3000 top variance genes in WD and PD differentiation.

Supplementary Data 5: Genes associated with ETNN bound, regular and super enhancers.

Supplementary Data 6: Erf-dependent genes associated with ETNN bound regular or super enhancers, LFC in ErfKO relative to WT PD.
