# Supplementary material for: The Fgf/Erf/NCoR1/2 repressive axis controls trophoblast cell fate
Source: Nat Commun. 2023 May 4;14:2559. doi: 10.1038/s41467-023-38101-8 (PMC10193302; doi:10.1038/s41467-023-38101-8)
Supplement: Supplementary file 9 — Reporting Summary [file 41467_2023_38101_MOESM9_ESM.pdf]

nature portfolio

Corresponding author(s): Paulina A. Latos

Last updated by author(s): Apr 6, 2023

Reporting Summary

Nature Portfolio wishes to improve the reproducibility of the work that we publish. This form provides structure for consistency and transparency in reporting. For further information on Nature Portfolio policies, see our Editorial Policies and the Editorial Policy Checklist.

Statistics

For all statistical analyses, confirm that the following items are present in the figure legend, table legend, main text, or Methods section.

n/a	Confirmed	
☐	☒	The exact sample size (*n*) for each experimental group/condition, given as a discrete number and unit of measurement
☐	☒	A statement on whether measurements were taken from distinct samples or whether the same sample was measured repeatedly
☐	☒	The statistical test(s) used AND whether they are one- or two-sided<br>*Only common tests should be described solely by name; describe more complex techniques in the Methods section.*
☒	☐	A description of all covariates tested
☐	☒	A description of any assumptions or corrections, such as tests of normality and adjustment for multiple comparisons
☒	☐	A full description of the statistical parameters including central tendency (e.g. means) or other basic estimates (e.g. regression coefficient) AND variation (e.g. standard deviation) or associated estimates of uncertainty (e.g. confidence intervals)
☒	☐	For null hypothesis testing, the test statistic (e.g. *F*, *t*, *r*) with confidence intervals, effect sizes, degrees of freedom and *P* value noted<br>*Give P values as exact values whenever suitable.*
☒	☐	For Bayesian analysis, information on the choice of priors and Markov chain Monte Carlo settings
☒	☐	For hierarchical and complex designs, identification of the appropriate level for tests and full reporting of outcomes
☒	☐	Estimates of effect sizes (e.g. Cohen's *d*, Pearson's *r*), indicating how they were calculated

Our web collection on statistics for biologists contains articles on many of the points above.

Software and code

Policy information about availability of computer code

Data collection	only free and publicly available software was used: bbduk (38.86), fastQC (0.11.9), STAR (2.7.3a), htseq (version 0.11.2); ChIPseq: CUTADAPT (2.8), bowtie 2 (2.3.5.1), samtools (1.10)
Data analysis	R (4.1), RStudio (1.4.1717), ggplot2 (3.3.3), pheatmap (1.0.12), Eulerr (6.1.0), UpSetR (1.4.0), clusterProfiler (4.0.5), Gprofiler2 (0.2.1), biomaRt (2.48.3), python (3.8), deeptools (3.5.0); RNAseq: DESeq2 (1.24.0), BisqueRNA (1.0.5), cluster (2.1.4), dendextend (1.16.0), factoextra (1.0.7); ChIPseq: MACS2 (2.2.7.1), ChIPpeakAnno (3.26.0), ChIPseeker (1.26.0), DiffBind (3.2.3), ROSE (2018-11-27)

For manuscripts utilizing custom algorithms or software that are central to the research but not yet described in published literature, software must be made available to editors and reviewers. We strongly encourage code deposition in a community repository (e.g. GitHub). See the Nature Portfolio guidelines for submitting code & software for further information.

Data

Policy information about availability of data

All manuscripts must include a data availability statement. This statement should provide the following information, where applicable:

- Accession codes, unique identifiers, or web links for publicly available datasets
- A description of any restrictions on data availability
- For clinical datasets or third party data, please ensure that the statement adheres to our policy

The raw and processed next-generation sequencing datasets were deposited at the NCBI Gene Expression Omnibus (GEO) repository under accession number: GSE199024, the proteomics data have been deposited to the ProteomeXchange Consortium via PRIDE partner repository with the dataset identifier PXD037892

March 2021

Field-specific reporting

Please select the one below that is the best fit for your research. If you are not sure, read the appropriate sections before making your selection.

☒ Life sciences ☐ Behavioural & social sciences ☐ Ecological, evolutionary & environmental sciences

For a reference copy of the document with all sections, see nature.com/documents/nr-reporting-summary-flat.pdf

Life sciences study design

All studies must disclose on these points even when the disclosure is negative.

Sample size	We did not perform sample size calculation, but used commonly applicable replicate numbers. For ChIPseq and RNAseq experiments n=2 and n=4(-8) respectively, for each condition, replicates were biological with independent KO cell lines where applicable and independent culturing for at least 5 passages where no independent KO lines were available.
Data exclusions	no data were excluded
Replication	Genome wide approaches were replicated as stated above, for all other experiments n is given in the specific section
Randomization	n/a
Blinding	n/a

Reporting for specific materials, systems and methods

We require information from authors about some types of materials, experimental systems and methods used in many studies. Here, indicate whether each material, system or method listed is relevant to your study. If you are not sure if a list item applies to your research, read the appropriate section before selecting a response.

Materials & experimental systems

n/a	Involved in the study	
☐	☒	Antibodies
☐	☒	Eukaryotic cell lines
☒	☐	Palaeontology and archaeology
☒	☐	Animals and other organisms
☒	☐	Human research participants
☒	☐	Clinical data
☒	☐	Dual use research of concern

Methods

n/a	Involved in the study	
☐	☒	ChIP-seq
☒	☐	Flow cytometry
☒	☐	MRI-based neuroimaging

Antibodies

Antibodies used	anti-Ncor1 (Abcam, ab3482), anti-Ncor2 (Abcam, ab5802), anti-Tbl1x (Proteintech, 13540-1-AP), anti-H3K27ac (Diagenode, C15410196), anti-H3K4me3 (Diagenode, C15410003), anti-V5 Agarose Affinity Gel (Sigma-Aldrich, A7345), anti-Erf (Santa Cruz, sc-398269), anti-Flag (F1804, Sigma Aldrich), anti-V5 (Sigma Aldrich, V8012), anti-Hdac3 (Abcam, ab7030), anti-Sox2 (R&D, AF2018), anti-Tubulin (Abcam, ab6160), anti-Erk1/2 (Cell Signalling, 4695), anti-phosphoErk1/2 (Cell Signalling, 9106), anti-LaminB1 (Santa Cruz, sc-374015), anti-rabbit IgG(L)-HRPP (Dianova, 211-032-171), anti-mouse IgG(L)-HRP (Dianova, 115-035-174)
Validation	All antibodies (see above) were commercially available and widely used

Eukaryotic cell lines

Policy information about cell lines

Cell line source(s)	TS EGFP line was a kind gift of Dr Janet Rossant and the parental cell lines for the newly established TSCs in this study: Tbl1x KO and ErfV5 KI were directly generated in the TS EGFP line; 2 independent ErfKO cell lines were generated from the ErfV5 KI TSCs
Authentication	Authentification was performed based on marker expression for all lines and PCR genotyping, qPCR and Western blot for the KO and rescue cell lines
Mycoplasma contamination	All cell lines tested negative for mycoplasma
Commonly misidentified lines (See ICLAC register)	none

ChIP-seq

Data deposition

- [x] Confirm that both raw and final processed data have been deposited in a public database such as GEO.
- [x] Confirm that you have deposited or provided access to graph files (e.g. BED files) for the called peaks.

Data access links<br>*May remain private before publication.*	https://www.ncbi.nlm.nih.gov/geo/query/acc.cgi?acc=GSE199024
Files in database submission	Merged_128605_TTCCTCCTTTGCTTGC.bam Merged_128606_TGCTTGCTTTGCTTGC.bam Merged_128607_GGTGATGATTGCTTGC.bam Merged_128608_AACCTACGTTGCTTGC.bam Merged_128609_GGATCTGATTGCTTGC.bam Merged_128610_TCGACCGACCAAGTGC.bam Merged_128611_AAGCGACTTTGCTTGC.bam Merged_128612_AAGCGTTCTTGCTTGC.bam Merged_128613_AAGGCGTATTGCTTGC.bam Merged_128614_CTGATGAGTTGCTTGC.bam Merged_128615_GCAGAAGATTGCTTGC.bam Merged_128616_AATCCAGCTTGCTTGC.bam Merged_128617_TTCCTCCTGAGAGGTT.bam Merged_128618_TGCTTGCTGAGAGGTT.bam Merged_128619_GGTGATGAGAGAGGTT.bam Merged_128620_AACCTACGGAGAGGTT.bam Merged_128621_GGATCTGAGAGAGGTT.bam Merged_128622_TGATCACGGAGAGGTT.bam Merged_128623_AAGCGACTGAGAGGTT.bam Merged_128624_AAGCGTTCGAGAGGTT.bam Merged_128625_AAGGCGTAGAGAGGTT.bam Merged_128626_CTGATGAGGAGAGGTT.bam Merged_128627_GCAGAAGAGAGAGGTT.bam Merged_128628_AATCCAGCGAGAGGTT.bam Merged_128629_TTCCTCCTACCTGGTT.bam Merged_128630_TGCTTGCTACCTGGTT.bam Merged_128631_GGTGATGAACCTGGTT.bam Merged_128632_AACCTACGACCTGGTT.bam Merged_128633_GGATCTGAACCTGGTT.bam Merged_128634_TGATCACGACCTGGTT.bam Merged_128635_AAGCGACTACCTGGTT.bam Merged_128636_AAGCGTTCACCTGGTT.bam Merged_128637_AAGGCGTAACCTGGTT.bam Merged_128638_CTGATGAGACCTGGTT.bam Merged_128639_GCAGAAGAACCTGGTT.bam Merged_128640_AATCCAGCACCTGGTT.bam Merged_128653_TTCCTCCTCGGAACAA.bam Merged_128654_TGCTTGCTCGGAACAA.bam Merged_128655_GGTGATGACGGAACAA.bam Merged_128656_AACCTACGCGGAACAA.bam Merged_128657_ATCGTGAGCTTACGGC.bam Merged_128658_TGATCACGCGGAACAA.bam Merged_128659_AAGCGACTCGGAACAA.bam Merged_128660_AAGCGTTCCGGAACAA.bam Merged_128661_AAGGCGTACGGAACAA.bam Merged_128662_CTGATGAGCGGAACAA.bam Merged_128663_GCAGAAGACGGAACAA.bam Merged_128664_AATCCAGCCGGAACAA.bam Merged_128665_TTCCTCCTGGTAAGCT.bam Merged_128666_TGCTTGCTGGTAAGCT.bam Merged_128667_GGTGATGAGGTAAGCT.bam Merged_128668_AACCTACGGGTAAGCT.bam Merged_128669_GGATCTGAGGTAAGCT.bam Merged_128670_TGATCACGGGTAAGCT.bam Merged_128671_AAGCGACTGGTAAGCT.bam Merged_128672_AAGCGTTCGGTAAGCT.bam Merged_128673_AAGGCGTAGGTAAGCT.bam Merged_128674_CTGATGAGGGTAAGCT.bam Merged_128675_GCAGAAGAGGTAAGCT.bam Merged_128676_AATCCAGCGGTAAGCT.bam Merged_128677_TTCCTCCTTGTGGCAT.bam Merged_128678_TGCTTGCTTGTGGCAT.bam Merged_128679_GGTGATGATGTGGCAT.bam Merged_128680_AACCTACGTGTGGCAT.bam Merged_128681_GGATCTGATGTGGCAT.bam Merged_128682_TGATCACGTGTGGCAT.bam Merged_128683_AAGCGACTTGTGGCAT.bam Merged_128684_AAGCGTTCTGTGGCAT.bam Merged_128685_AAGGCGTATGTGGCAT.bam Merged_128686_CTGATGAGTGTGGCAT.bam Merged_128687_GCAGAAGATGTGGCAT.bam Merged_128688_AATCCAGCTGTGGCAT.bam Merged_128689_TTCCTCCTACTACGGA.bam Merged_128690_TGCTTGCTACTACGGA.bam Merged_128691_GGTGATGAACTACGGA.bam Merged_128692_AACCTACGACTACGGA.bam Merged_128693_GGATCTGAACTACGGA.bam Merged_128694_TGATCACGACTACGGA.bam Merged_128695_AAGCGACTACTACGGA.bam Merged_128696_AAGCGTTCACTACGGA.bam Merged_128697_AAGGCGTAACTACGGA.bam Merged_128698_CTGATGAGACTACGGA.bam Merged_128699_GCAGAAGAACTACGGA.bam Merged_128700_AATCCAGCACTACGGA.bam 163190_S1_R1_001.fastq.gz 163191_S2_R1_001.fastq.gz 163192_S3_R1_001.fastq.gz 163193_S4_R1_001.fastq.gz 163194_S5_R1_001.fastq.gz 163195_S6_R1_001.fastq.gz 163196_S7_R1_001.fastq.gz 163197_S8_R1_001.fastq.gz 163198_S9_R1_001.fastq.gz 163199_S10_R1_001.fastq.gz 163200_S11_R1_001.fastq.gz 163201_S12_R1_001.fastq.gz 163202_S13_R1_001.fastq.gz 163203_S14_R1_001.fastq.gz 163204_S15_R1_001.fastq.gz 163205_S16_R1_001.fastq.gz 163190_S1_R2_001.fastq.gz 163191_S2_R2_001.fastq.gz 163192_S3_R2_001.fastq.gz 163193_S4_R2_001.fastq.gz 163194_S5_R2_001.fastq.gz 163195_S6_R2_001.fastq.gz 163196_S7_R2_001.fastq.gz 163197_S8_R2_001.fastq.gz 163198_S9_R2_001.fastq.gz 163199_S10_R2_001.fastq.gz 163200_S11_R2_001.fastq.gz 163201_S12_R2_001.fastq.gz 163202_S13_R2_001.fastq.gz 163203_S14_R2_001.fastq.gz 163204_S15_R2_001.fastq.gz 163205_S16_R2_001.fastq.gz 101602_CTTGTA_CE0NEANXX_4_20191021B_20191021.bam V5_TSGFP-ErfV5_PD_129469_GATCAG.bam 101601_GGCTAC_CE0NEANXX_4_20191021B_20191021.bam V5_TSGFP_PD_129472_CTTGTA.bam Erf_V5_V5PD_101602_129469_GL.bw H3K27ac_ErfKO_PD_128634_128636.bw H3K27ac_ErfKO_SR_128633_128635.bw H3K27ac_ErfRescue_PD_128638_128640.bw H3K27ac_ErfRescue_SR_128637_128639.bw H3K27ac_V5PD_128631_128632.bw H3K27ac_V5SR_128629_128630.bw H3K4me3_ErfKO_128621_128623.bw H3K4me3_ErfKO_PD_128622_128624.bw H3K4me3_ErfRescue_PD_128626_128628.bw H3K4me3_ErfRescue_SR_128625_128627.bw H3K4me3_V5PD_128619_128620.bw H3K4me3_V5SR_128617_128618.bw Ncor1_ErfKO_PD_128694_128696.bw Ncor1_ErfKO_SR_128693_128695.bw Ncor1_ErfRescue_PD_128698_128700.bw Ncor1_ErfRescue_SR_128697_128699.bw Ncor1_V5PD_128691_128692.bw Ncor1_V5SR_128689_128690.bw Ncor2_ErfKO_PD_128682_128684.bw Ncor2_ErfKO_SR_128681_128683.bw Ncor2_ErfRescue_PD_128686_128688.bw Ncor2_ErfRescue_SR_128685_128687.bw Ncor2_V5PD_128679_128680.bw Ncor2_V5SR_128677_128678.bw Tbl1x_ErfKO_PD_163195_163197.bw Tbl1x_ErfKO_SR_163194_163196.bw Tbl1x_V5PD_163192_163193.bw Tbl1x_V5SR_163190_163191.bw Erf_CoV5_WT_PD_IDR_CPA.bed H3K27ac_ErfKO_PD_IDR_CPA.bed

	H3K27ac_ErfKO_SR_IDR_CPA.bed H3K27ac_ErfRescue_PD_IDR_CPA.bed H3K27ac_ErfRescue_SR_IDR_CPA.bed H3K27ac_WT_PD_IDR_CPA.bed H3K27ac_WT_SR_IDR_CPA.bed H3K4me3_ErfKO_PD_IDR_CPA.bed H3K4me3_ErfKO_SR_IDR_CPA.bed H3K4me3_ErfRescue_PD_IDR_CPA.bed H3K4me3_ErfRescue_SR_IDR_CPA.bed H3K4me3_WT_PD_IDR_CPA.bed H3K4me3_WT_SR_IDR_CPA.bed Ncor1_ErfKO_PD_IDR_CPA.bed Ncor1_ErfKO_SR_IDR_CPA.bed Ncor1_ErfRescue_PD_IDR_CPA.bed Ncor1_ErfRescue_SR_IDR_CPA.bed Ncor1_WT_PD_IDR_CPA.bed Ncor1_WT_SR_IDR_CPA.bed Ncor2_ErfKO_PD_IDR_CPA.bed Ncor2_ErfKO_SR_IDR_CPA.bed Ncor2_ErfRescue_PD_IDR_CPA.bed Ncor2_ErfRescue_SR_IDR_CPA.bed Ncor2_WT_PD_IDR_CPA.bed Ncor2_WT_SR_IDR_CPA.bed Tbl1x_ErfKO_PD_IDR_CPA.bed Tbl1x_ErfKO_SR_IDR_CPA.bed Tbl1x_WT_PD_IDR_CPA.bed Tbl1x_WT_SR_IDR_CPA.bed
Genome browser session (e.g. UCSC)	N/A

Methodology

Replicates	2 biological replicates were generated for each ChIP and input, where available independent KO clones were used to replicate data.
Sequencing depth	currently not included, but available as separate file
Antibodies	anti-Ncor1 (Abcam, ab3482), anti-Ncor2 (Abcam, ab5802), anti-Tbl1x (Proteintech, 13540-1-AP), anti-H3K27ac (Diagenode, C15410196), anti-H3K4me3 (Diagenode, C15410003), anti-V5 Agarose Affinity Gel (Sigma-Aldrich, A7345).
Peak calling parameters	Peaks were called using MACS2 (2.2.7.1) with a p-value cutoff of 0.1. High confidence peaks were generated by IDR (1.2), using a cut-off of FDR<0.05 across all replicates via the ChIPpeakAnno package (3.26.0).
Data quality	Replicate peaks were called separately and filtered for IDR < 0.05 (see above).
Software	CUTADAPT (2.8), bowtie 2 (2.3.5.1), samtools (1.10), MACS2 (2.2.7.1), ChIPpeakAnno (3.26.0), ChIPseeker (1.26.0), DiffBind (3.2.3), ROSE (2018-11-27)
